# Four major psychiatric disorders in childhood and early adulthood and siblings’ subsequent socioeconomic status: a nationwide register study

**DOI:** 10.1007/s00127-025-02997-y

**Published:** 2025-09-27

**Authors:** Wen Yang, Kaisla Komulainen, Ripsa Niemi, Mai Gutvilig, Petri Böckerman, Marko Elovainio, Christian Hakulinen

**Affiliations:** 1 Department of Psychology, Faculty of Medicine, University of Helsinki, Helsinki, Finland; 2 Finnish Institute for Health and Welfare, Helsinki, Finland; 3 School of Business and Economics, University of Jyväskylä, Jyväskylä, Finland; 4 Labour Institute for Economic Research LABORE, Helsinki, Finland; 5 IZA Institute of Labor Economics, Bonn, Germany; 6 University of Helsinki, P.O. Box 21, 00014 Helsinki, Finland

**Keywords:** Siblings, Major psychiatric disorders, Socioeconomic status, Childhood and early adulthood

## Abstract

**Purpose:**

Previous studies document the clustering of major psychiatric disorders (MPDs) – schizophrenia, bipolar disorder, depression, and anxiety – among siblings. Few studies have, however, examined whether MPDs during childhood and early adulthood are associated with siblings’ future socioeconomic status (SES).

**Methods:**

This cohort study included 57,537 full siblings, 4653 paternal, and 5053 maternal half-siblings of individuals with MPDs (affected probands) born in Finland between 1970 and 1990. We defined the reference groups as identical types of siblings of individuals without an MPD diagnosis (unaffected probands) and followed both siblings of the affected and unaffected probands until December 31, 2020. MPDs diagnosed among the affected probands at ages 5–25 was obtained from the Finnish Care Register. Their siblings’ SES was measured based on employment status, annual disposable income, and educational achievement. Logistic regression, median regression, and generalized estimating equations (GEE) were used to estimate the associations.

**Results:**

Compared to the siblings of the unaffected probands, the odds of unemployment at the end of follow-up were 35% higher (95% CI: 1.31–1.39) in full siblings of affected probands with an MPD. Full siblings of affected probands were also more likely not to achieve a higher education level (aOR: 1.28, 95% CI 1.24–1.31). The median annual disposable income was 1255.9 EUR lower (95% CI: –1385.6, –1126.3) in full siblings of affected probands. Similar but weaker associations were observed in maternal and paternal half-siblings. Results from GEE models using repeated measurements of income and unemployment were similar.

**Conclusion:**

Our findings suggest that the socioeconomic consequences associated with MPDs extend to siblings.

**Supplementary Information:**

The online version contains supplementary material available at 10.1007/s00127-025-02997-y.

## Background

The burden of mental disorders is considerable among children and young people, particularly in high-income countries [1–4]. Depressive disorders, schizophrenia, and bipolar disorder are associated with the highest burden both at the individual and societal levels [1, 3]. In addition, despite receiving less attention [5, 6], anxiety disorders are the most prevalent mental disorders in adolescence [3]. The long-term consequences of these major psychiatric disorders (MPDs) in young people are profound. For example, several studies have reported associations of depression, bipolar disorder and schizophrenia with poor socioeconomic status (SES), typically measured using educational achievement, employment, and income [7–11]. Previous research has also shown that major psychiatric disorders can cluster within families and pass across generations, with strong associations between siblings [12–15].

Sibling relationships are enduring and uniquely affected by common genetic, social, and cultural backgrounds [16]. Siblings have a significant direct or indirect role in each other’s development, with either positive or negative influence [17, 18]. Younger siblings can benefit from older siblings’ caregiving, such as emotional support, but can also develop low school performance, for instance, if influenced by their co-siblings’ negative characteristics [19]. Previous findings also suggest impeded psychosocial development among children who have a sibling with a mental disorder [20–22]. The consequences of growing up with a sibling with a mental disorder may extend further into later life stages. Notably, exposure to a sibling’s mental disorder in childhood or adolescence could significantly influence socioeconomic outcomes among individuals as they mature into adulthood.

To date, relatively few studies have directly examined whether major psychiatric disorders in childhood and early adulthood are associated with the socioeconomic outcomes of siblings [22–24]. A Danish longitudinal study [22] compared socioeconomic outcomes between adult siblings of patients with bipolar disorder and matched general population controls, observing lower educational achievement and other SES indicators among siblings of patients with bipolar disorder. However, it remains uncertain whether similar findings prevail in other major mental disorders, especially when they are diagnosed at a younger age.

Leveraging nationwide Finnish register data, we examined the associations between having a proband with a major psychiatric disorder (MPD; schizophrenia spectrum disorder, bipolar disorder, depressive disorder, or anxiety disorder) diagnosed at age 5–25 as a sibling and subsequent educational achievement, employment, and income. The secondary aim was to examine whether the associations differ across demographic characteristics of siblings and their probands.

## Methods

### Data sources

For this population-based cohort study, we obtained data from FOLK modules of Statistics Finland and the Finnish Care Register (FCR), combined using pseudonymized unique personal identification numbers assigned to all Finnish citizens. FOLK modules of Statistics Finland contain information on socioeconomic status, residence, migration, and other demographics. The FCR contains comprehensive information on healthcare contacts, including inpatient and outpatient records, as well as emergency department visits. Detailed description of used registers was shown in the Supplementary material.

The study plan was approved by the Ethics Committee of the Finnish Institute of Health and Welfare (THL/184/6.02.01/2023§ 933), and data were linked with the permission of Statistics Finland (TK-53-1696-16) and the Finnish Institute of Health and Welfare. According to Finnish law, register-based research does not require informed consent. This study followed the Strengthening the Reporting of Observational Studies in Epidemiology (STROBE) reporting guideline.

## Study population and exposure

We aimed to align cohort selection with the availability of national registers. Diagnoses of MPD were obtained from Finnish Care Register (FCR), which began to include hospital admission records in 1970. To ensure higher data validity, we decided to include diagnoses from 1980 onward, selecting individuals born between 1975 and 1985. This allowed for ascertainment of MPD diagnoses between 1980 and 2010, capturing diagnoses made between ages 5 and 25. Thus, the initial cohort included 704,084 individuals born in Finland between January 1, 1975, and December 31, 1985. We excluded individuals who had no biological siblings or who were twins as twins may have higher genetic similarity and greater environmental overlap than singleton sibling pairs, they may have a different risk profile, as well as those who emigrated or died before age 5. First, we selected individuals with any first-ever diagnosed MPD at 5–25 years old according to the *International Statistical Classification of Diseases*,* Tenth Revision* (ICD-10), *and the previous revisions* (ICD-8 and ICD-9). The MPDs included schizophrenia spectrum disorders, bipolar disorder, depressive disorders, and anxiety disorders (F20-29, F30-31, F32-33, F34.1, F39, F40-48 in ICD-10; 295, 297, 298, 301.0, 301.2, 296, 298, 300 in ICD-8 and ICD-9) diagnosed between ages 5–25, irrespective of psychiatric comorbidity. The baseline in our study was defined as the date of the first identified diagnosis of any MPD.

We then matched everyone with an MPD to a maximum of 10 randomly selected general population controls without an MPD diagnosis based on birth year, sex, and county of residence at baseline. Similarly, we matched everyone with a specific psychiatric disorder to a maximum of 10 randomly selected general population controls without that specific disorder. This exact matching procedure was applied separately to full sibling, maternal half-sibling, and paternal half-sibling cohorts. We refer to individuals with a first-ever diagnosed MPD as “affected probands” and their matched general population controls as “unaffected probands”. Finally, we identified the affected and unaffected probands’ full siblings and half-siblings (paternal and maternal, separately) born in Finland between January 1, 1970, and December 31, 1990. This allowed the inclusion of both older (born before 1975) and younger (born after 1985) siblings of probands, increasing the sample size and statistical power especially for analyses involving half-siblings. We excluded siblings who had a diagnosis of intellectual disability (ICD-10 F70-79, ICD-9 317–319, ICD-8 310–314) or autism spectrum disorder (ICD-10 F84.0, ICD-8 and 9 299), as well as those who had emigrated or died before baseline (Figure S1–S3). A total of 57,537 full siblings, 5053 maternal half-siblings, and 4653 paternal half-siblings of affected probands with MPDs and their corresponding 537,057 full siblings, 15,334 maternal half-siblings, 14,835 paternal half-siblings of unaffected probands were eligible at baseline. Socioeconomic outcomes among siblings of the affected and unaffected probands were followed from the baseline until December 31, 2020.

## Socioeconomic outcomes

Socioeconomic outcomes among siblings of affected and unaffected probands were measured using data on educational achievement, annual employment status, and personal disposable income (measured in EUR).

Educational achievement was defined as not having a higher education degree (coded as 1) and having a higher education degree (coded as 0) at the end of follow-up. During the study period, all children in Finland were required to begin compulsory education at age seven on average, comprising nine years of primary and lower secondary education. At the end of mandatory school, students were assessed for eligibility for upper secondary school or vocational education, which takes an average of three years to complete. Afterward, students can apply for further university studies, i.e., higher education [25].

Annual employment status (unemployed coded as 1, employed coded as 0) was measured as the employment status during the last week of each calendar year. Individuals who were wage and salary earners or self-employed were defined as employed, and others were classified as unemployed.

Personal disposable income was measured annually as a sum of wage and salary earnings, self-employment income, and social security benefits. It was deflated to the base year 2015 using the consumer price index of Statistics Finland to ensure the comparability of income measures over the observation period. To mitigate the influence of outliers at the upper end of the income distribution, we top-coded personal disposable income at the 99th percentile [26].

## Covariates

We included data on the following covariates from siblings of the affected and unaffected probands (i.e., siblings whose SES outcomes we followed): sex, age (continuous variable), second-generation migration status, residential location at baseline, individual’s own MPD diagnosis before baseline, paternal and maternal highest education level at baseline, paternal and maternal history of mental disorders before baseline, and paternal and maternal age at the time of the individual’s birth, and the square of paternal and maternal age at birth of siblings of the affected and unaffected probands (continuous variable). Individuals were considered second-generation migrants if they were Finnish born with two parents born outside Finland. Residential location was classified into four categories: Helsinki Central Area, Northern and Eastern Finland, Western Finland, and Southern Finland (including Åland). The parents’ highest education level at baseline was categorized as compulsory education, upper secondary education, or higher education; missing values were coded into a separate category. Paternal and maternal history of mental disorders (yes/no) before the baseline was obtained from the FCR using ICD-10 F-chapter codes F10-F99.

### Statistical analysis

First, we estimated the associations between having an affected proband and SES at the end of follow-up (end-point analysis) using logistic regression models for the binary outcomes, i.e., educational achievement and employment status and median regression models for the continuous outcome, i.e., personal disposable income (EUR). Individuals with missing SES data at the end of follow-up were excluded from these analyses.

Second, to estimate the associations between having an affected proband and annual employment and disposable income over the follow-up period (longitudinal analysis), we used generalized estimating equations (GEE). Here, we estimated the odds of unemployment and the average marginal effects for disposable income [27], i.e., the absolute income differences between siblings of the affected and unaffected probands using the repeated measurements of employment and income each year since baseline. All GEE models assumed an exchangeable correlation structure. The logit and log link functions were applied separately to employment and income outcomes. Since data on income and employment status were only available from 1987 onwards, these two outcomes for individuals whose affected probands received an MPD diagnosis before 1987—and for their corresponding referents—were measured beginning in 1987. If an individual did not have SES data for a specific year after 1987, the corresponding comparators were not included in the analysis.

In all analyses, we first estimated unadjusted models (i.e., crude models without any adjustments), and then adjusted for the following characteristics of the siblings whose SES outcomes we followed: age at baseline, second-generation migration status, residential location at baseline, individual’s own MPD diagnosis before baseline, paternal and maternal highest education level at baseline, paternal and maternal history of mental disorders before baseline, and paternal and maternal age at the time of the individual’s birth. In addition, we included the square of paternal and maternal age to account for the potentially U-shaped association of parental age with rates of MPDs among the probands and SES outcomes among their siblings.

We then repeated the analyses stratifying by siblings’ sex, and age of their probands at baseline (5–14, 15–19, and 20–25 years). As an additional analysis, we redefined personal disposable to include only wage and salary earnings plus self-employment income, excluding social security benefits, because under the Finnish social security system, residents can receive various benefits when they cannot participate in regular working life [28].

In families with more than one case with MPD, we defined a matching cluster that included each affected proband, their own matched unaffected proband and both sets of specific siblings [29]. Three datasets were then created to analyze the full, maternal, and paternal half-siblings. This led to a dependency in the data, and some individuals will be duplicated. To account for the relatedness of observations, the robust sandwich estimator of standard errors was used in the logistic regression, median regression, and GEE models [30].

Data management was performed using Stata/MP 17.0 (StataCorp, College Station, Texas, USA) and R version 4.2.2 (R Foundation for Digital Computing, Vienna, Austria). Data analysis was performed using Stata/MP 17.0. All statistical tests are two-sided; a *P*-value below 0.05 was considered significant.

## Results

### Characteristics of the study sample

The median age (interquartile range, IQR) at baseline was 20 years (16–24) for full siblings, 17 years (12–26) for maternal half-siblings, and 18 years (12–26) for paternal half-siblings of the affected and unaffected probands (Table 1). At baseline, full siblings, maternal and paternal half siblings of affected probands with MPDs were more likely to have a parent with a history of mental disorders than siblings of unaffected probands (*P* < 0.001 for all). Descriptive characteristics of siblings of the affected and unaffected probands eligible at baseline are reported by specific MPD diagnoses in Tables S1-S4.

**Table 1:** Descriptive characteristics of observations of siblings of the affected and unaffected probands with any diagnosed MPD

	Full siblings		Maternal half-siblings		Paternal half-siblings	
	Unaffected	Affected	Unaffected	Affected	Unaffected	Affected
Total (N)	537,057	57,537	15,334	5,053	14,835	4,653
*Sex, n (%)*						
Men	273,245 (50.9%)	28,953 (50.3%)	7,796 (50.8%)	2,516 (49.8%)	7,626 (51.4%)	2,320 (49.9%)
Women	263,812 (49.1%)	28,584 (49.7%)	7,538 (49.2%)	2,537 (50.2%)	7,209 (48.6%)	2,333 (50.1%)
Age at baseline, years^*^	20.0 (16.0–24.0)	20.0 (16.0–24.0)	17.0 (12.0–26.0)	17.0 (11.0–26.0)	18.0 (12.0–26.0)	18.0 (12.0–26.0)
*Migration status, n (%)*						
Finnish born	536,830 (100.0%)	57,524 (100.0%)	15,330 (100.0%)	NA	14,826 (99.9%)	NA
Second-generation migrant	227 (0.0%)	13 (0.0%)	4 (0.0%)	NA	9 (0.1%)	NA
Any MPD diagnosis before baseline, yes, n (%)	15,864 (3.0%)	6,151 (10.7%)	718 (4.7%)	444 (8.8%)	616 (4.2%)	375 (8.1%)
*Residential region at baseline, n (%)*						
Helsinki Central Area	146,363 (27.3%)	16,965 (29.5%)	4,068 (26.5%)	1,468 (29.1%)	4,501 (30.3%)	1,490 (32.0%)
Southern Finland	105,125 (19.6%)	11,354 (19.7%)	3,801 (24.8%)	1,227 (24.3%)	3,499 (23.6%)	1,074 (23.1%)
Western Finland	129,204 (24.1%)	13,393 (23.3%)	3,686 (24.0%)	1,180 (23.4%)	3,400 (22.9%)	1,060 (22.8%)
Northern and Eastern Finland	155,711 (29.0%)	15,776 (27.4%)	3,578 (23.3%)	1,117 (22.1%)	3,261 (22.0%)	973 (20.9%)
Missing	654 (0.1%)	49 (0.1%)	201 (1.3%)	61 (1.2%)	174 (1.2%)	56 (1.2%)
**Characteristics of probands**						
*Sex, n (%)*						
Men	285,170 (53.1%)	30,002 (52.1%)	8,603 (56.1%)	2,831 (56.0%)	8,041 (54.2%)	2,584 (55.5%)
Women	251,887 (46.9%)	27,535 (47.9%)	6,731 (43.9%)	2,222 (44.0%)	6,794 (45.8%)	2,069 (44.5%)
*Age of probands at baseline (years), n (%)*						
5–14	32,020 (5.9%)	3,222 (5.6%)	1,776 (11.6%)	596 (11.8%)	1,409 (9.5%)	478 (10.3%)
15–19	205,618 (38.3%)	21,406 (37.2%)	5,854 (38.2%)	1,877 (37.1%)	5,760 (38.8%)	1,783 (38.3%)
20–25	299,419 (55.8%)	32,909 (57.2%)	7,704 (50.2%)	2,580 (51.1%)	7,666 (51.7%)	2,392 (51.4%)
**Characteristics of parents**						
Paternal age at birth of siblings, years^*^	29.0 (26.0–33.0)	29.0 (25.0–33.0)	28.0 (24.0–32.0)	28.0 (24.0–32.0)	28.0 (24.0–33.0)	28.0 (24.0–33.0)
Maternal age at birth of siblings, years^*^	27.0 (24.0–31.0)	27.0 (23.0–31.0)	26.0 (21.0–30.0)	26.0 (21.0–30.0)	25.0 (21.0–30.0)	25.0 (21.0–29.0)
*Paternal highest education level at baseline, n (%)*						
Lower secondary or less	144,929 (27.0%)	16,726 (29.1%)	4,946 (32.3%)	1,700 (33.6%)	4,928 (33.2%)	1,625 (34.9%)
Upper secondary	191,929 (35.7%)	20,553 (35.7%)	5,897 (38.5%)	2,059 (40.7%)	5,634 (38.0%)	1,819 (39.1%)
Post-secondary or tertiary	173,304 (32.3%)	15,839 (27.5%)	2,160 (14.1%)	540 (10.7%)	2,820 (19.0%)	662 (14.2%)
Missing	26,895 (5.0%)	4,419 (7.7%)	2,331 (15.2%)	754 (14.9%)	1,453 (9.8%)	547 (11.8%)
*Maternal highest education level at baseline, n (%)*						
Lower secondary or less	120,092 (22.4%)	15,152 (26.3%)	5,558 (36.2%)	1,967 (38.9%)	4,227 (28.5%)	1,498 (32.2%)
Upper secondary	223,761 (41.7%)	23,953 (41.6%)	6,703 (43.7%)	2,197 (43.5%)	6,316 (42.6%)	1,987 (42.7%)
Post-secondary or tertiary	180,891 (33.7%)	16,597 (28.8%)	2,377 (15.5%)	599 (11.9%)	3,433 (23.1%)	900 (19.3%)
Missing	12,313 (2.3%)	1,835 (3.2%)	696 (4.5%)	290 (5.7%)	859 (5.8%)	268 (5.8%)
Paternal history of mental disorder before the baseline, yes, n (%)	41,954 (7.8%)	8,852 (15.4%)	2,895 (18.9%)	1,096 (21.7%)	2,483 (16.7%)	1,212 (26.0%)
Maternal history of mental disorder before the baseline, yes, n (%)	34,319 (6.4%)	8,229 (14.3%)	2,028 (13.2%)	1,263 (25.0%)	1,734 (11.7%)	659 (14.2%)

^*^Median and IQR

NA: data unavailable due to very few observations (< 3)

Differences in baseline characteristics (i.e., from the date of the first diagnosis of any MPDs) were tested using the χ^2^ test for categorical variables and the Mann-Whitney U test for continuous variables, *P* < 0.001 for all

## Associations between having an affected proband and subsequent SES outcomes

Compared to full siblings of an unaffected proband, the full siblings of the affected probands with any MPD were more likely unemployed at the end of the follow-up (end-point analysis) (aOR = 1.35, 95% CI 1.31–1.39) (Table 2). Similarly, the median annual disposable income was 1255.9 EUR lower (95% CI −1385.6, −1126.3) in full siblings of affected probands with any MPD at the end of follow-up (Table 2). Full siblings of the affected probands with any MPD had 28% higher odds of not achieving a higher education degree (95%CI 1.24–1.31) after controlling potential confounders. The results were generally similar across the specific MPDs (Table 2). The largest differences in disposable income (a$\:\beta\:\:$= −1887.5, 95% CI, −2233.2, −1541.7), and odd ratios for unemployment (aOR = 1.60, 95% CI 1.49–1.71) at the end of follow-up were observed among full siblings of affected probands with schizophrenia spectrum disorders compared to full siblings of the corresponding unaffected probands. Mostly similar but weaker associations were observed in paternal and maternal half-siblings. For example, compared to the half-siblings of the unaffected probands, the adjusted odds of unemployment were 30% (95% CI 1.18–1.44) and 23% (95% CI 1.11–1.36) higher in maternal and paternal half-siblings of the affected proband with any MPD at the end of follow up (Table 2).

**Table 2:** Associations between having an affected proband and subsequent SES at the end of follow-up (End-point analysis)

	Full siblings		Maternal half-siblings		Paternal half-siblings	
	Crude	Adjusted	Crude	Adjusted	Crude	Adjusted
***Any MPD***						
**Education**	*N* = 386,360	*N* = 383,173	*N* = 13,740	*N* = 12,975	*N* = 13,047	*N* = 12,684
Not having a higher education degree	0.69 (0.67–0.70)	1.28 (1.24–1.31)	0.74 (0.67–0.82)	1.20 (1.08–1.33)	0.76 (0.69–0.84)	1.28 (1.24–1.31)
**Employment**	*N* = 354,626	*N* = 351,766	*N* = 12,655	*N* = 11,918	*N* = 12,105	*N* = 11,773
Unemployed	1.61 (1.56–1.66)	1.35 (1.31–1.39)	1.42 (1.30–1.56)	1.30 (1.18–1.44)	1.32 (1.20–1.45)	1.23 (1.11–1.36)
**Income (median**, EUR)^*^	*N* = 354,506	*N* = 351,762	*N* = 12,635	*N* = 11,918	*N* = 12,101	*N* = 11,773
Disposable income (top-coded)	−1956.4 ( −2087.8, −1825.1)	−1255.9 ( −1385.6, −1126.3)	−950.2 (−1385.4, −515.0)	−956.9 (−1392.5, −521.2)	−1116.9 (−1540.7, −693.1)	−879.1 (−1328.2, −430.1)
***Anxiety***						
**Education**	*N* = 255,997	*N* = 253,968	*N* = 11,261	*N* = 10,607	*N* = 10,994	*N* = 10,676
Not having a higher education degree	0.66 (0.64–0.68)	1.30 (1.26–1.35)	0.68 (0.61–0.76)	1.29 (1.15–1.44)	0.75 (0.67–0.83)	1.30 (1.26–1.35)
**Employment**	*N* = 235,072	*N* = 233,293	*N* = 10,350	*N* = 9,727	*N* = 10,113	*N* = 9,815
Unemployed	1.59 (1.54–1.65)	1.37 (1.32–1.42)	1.43 (1.29–1.58)	1.24 (1.12–1.38)	1.26 (1.13–1.40)	1.15 (1.03–1.29)
**Income**	*N* = 235,019	*N* = 233,307	*N* = 10,443	*N* = 9,727	*N* = 10,162	*N* = 9,815
Disposable income (top-coded)	−2078.7 (−2236.8, −1920.6)	−1187.6 ( −1351.5, −1023.8)	−1114.4 (−1593.6, −635.3)	−693.4 (−1159.9, −226.8)	−1203.2 (−1636.3, −770.1)	−878.1 (−1413.3, −342.9)
***Depression***						
**Education**	*N* = 208,709	*N* = 207,224	*N* = 8,783	*N* = 8,218	*N* = 8,645	*N* = 8,353
Not having a higher education degree	0.70 (0.67–0.72)	1.27 (1.22–1.31)	0.82 (0.73–0.93)	1.04 (0.91–1.19)	0.81 (0.72–0.91)	1.27 (1.22–1.31)
**Employment**	*N* = 191,650	*N* = 190,281	*N* = 8,185	*N* = 7,663	*N* = 8,122	*N* = 7,859
Unemployed	1.60 (1.54–1.66)	1.35 (1.30–1.41)	1.44 (1.29–1.62)	1.27 (1.13–1.44)	1.27 (1.13–1.43)	1.11 (0.98–1.26)
**Income**	*N* = 191,477	*N* = 190,284	*N* = 8,170	*N* = 7,663	*N* = 8,043	*N* = 7,859
Disposable income (top-coded)	−1957.2 ( −2131.4, −1783.0)	−1171.0 ( −1351.8, −990.3)	−1518.9 (−2018.6, −1019.2)	−786.0 (−1330.3, −241.8)	−1390.9 (−1925.1, −856.7)	−1051.4 (−1565.8, −537.0)
***Schizophrenia***						
**Education**	*N* = 65,815	*N* = 65,228	*N* = 2,765	*N* = 2,626	*N* = 2,781	*N* = 2,687
Not having a higher education degree	0.75 (0.71–0.80)	1.24 (1.16–1.32)	0.82 (0.66–1.03)	1.12 (0.88–1.42)	0.90 (0.73–1.11)	1.24 (1.16–1.32)
**Employment**	*N* = 60,047	*N* = 59,541	*N* = 2,593	*N* = 2,461	*N* = 2,520	*N* = 2,449
Unemployed	1.87 (1.74-2.00)	1.60 (1.49–1.71)	1.63 (1.32-2.00)	1.54 (1.24–1.92)	1.36 (1.09–1.68)	1.24 (0.99–1.56)
**Income**	*N* = 60,097	*N* = 59,541	*N* = 2,545	*N* = 2,461	*N* = 2,563	*N* = 2,449
Disposable income (top-coded)	−2411.4 ( −2752.9, −2069.9)	−1887.5 ( −2233.2, −1541.7)	−1746.0 (−2751.6, −740.4)	−1904.8 (−2880.9, −928.7)	−786.8 (−1831.9, 258.3)	−1342.8 (−2359.1, −326.4)
***Bipolar disorder***						
**Education**	*N* = 19,802	*N* = 19,656	*N* = 808	*N* = 748	*N* = 990	*N* = 953
Not having a higher education degree	0.76 (0.69–0.85)	1.26 (1.12–1.42)	0.90 (0.62–1.30)	1.22 (0.81–1.85)	1.06 (0.78–1.46)	1.26 (1.12–1.42)
**Employment**	*N* = 18,148	*N* = 18,018	*N* = 729	*N* = 681	*N* = 900	*N* = 872
Unemployed	1.78 (1.58–2.01)	1.52 (1.35–1.72)	1.62 (1.13–2.30)	1.86 (1.25–2.77)	1.61 (1.13–2.29)	1.24 (0.99–1.56)
**Income**	*N* = 18,226	*N* = 18,018	*N* = 738	*N* = 681	*N* = 887	*N* = 872
Disposable income (top-coded)	−1758.6 ( −2335.4, −1181.8)	−1659.9( −2314.3, −1005.6)	−139.2 (−2339.6, 2061.2)	−816.3 (−2661.2, 1028.6)	−1238.6 (−3377.3, 900.0)	−1143.6 (−3016.0, 728.8)

The reference group was the siblings of unaffected probands

Adjusted: sex, age, residential region at baseline, any MPD diagnosis in the siblings before baseline, paternal and maternal highest education level at baseline, paternal and maternal history of any mental disorders before baseline, paternal and maternal age at birth of siblings of the affected and unaffected probands and the square of paternal and maternal age

The estimates for education and employment are ORs and 95% CI

^*^The estimates for income are coefficient and 95%CI

The results were similar in GEE models (longitudinal analysis) using the repeated annual employment status and income measurements (Table 3). Compared to siblings of the unaffected probands, the odds for being unemployed were greater in full siblings (1.22, 95% CI 1.20–1.23) than in maternal half-siblings (1.17, 95% CI 1.12–1.22) or paternal half-siblings (1.14, 95% CI 1.09–1.19) of the affected probands with any MPD. A similar descending pattern was observed for income: −947.8 EUR (95% CI, −1018.0, −876.4) among full siblings, −472.8 EUR (95%CI, −687.8, −257.8) among maternal half-siblings, and − 490.2 EUR (95%CI, −728.5, −251.9) among paternal half-siblings. The largest disposable income gap (in EUR) was again observed between full siblings of the affected probands with schizophrenia spectrum disorders and corresponding unaffected controls (a$\:\beta\:$: −1512.8, 95% CI −1693.1, −1332.6). Similarly, the odds ratio for unemployment was the largest when comparing full siblings of affected probands with schizophrenia spectrum disorders to full siblings of the corresponding unaffected probands (aOR = 1.43, 95% CI: 1.39–1.47).

**Table 3:** Associations between having an affected proband and subsequent employment status and income over the observation period (Longitudinal analysis)

	Full siblings		Maternal half-siblings		Paternal half-siblings	
	Crude	Adjusted	Crude	Adjusted	Crude	Adjusted
***Any MPDs***						
**Employment**	*N* = 593,536	*N* = 588,646	*N* = 20,350	*N* = 19,171	*N* = 19,457	*N* = 18,908
Unemployed	1.31 (1.30–1.33)	1.22 (1.20–1.23)	1.27 (1.21–1.32)	1.17 (1.12–1.22)	1.19 (1.14–1.25)	1.14 (1.09–1.19)
**Income (mean**,** EUR)**	*N* = 593, 464	*N* = 588, 574	*N* = 20,346	*N* = 19,164	*N* = 19,451	*N* = 18,901
Disposable income (top-coded)	−1345.3 (−1429.3, −1261.2)	−947.8 (−1018.0, −876.4)	−1083 (−1362.1, −804.0)	−472.8 (−687.8, −257.8)	−843.1 (−1149.4, −536.7)	−490.2 (−728.5, −251.9)
Earnings^**^	−3025.1 (−3085.2, −2965)	−1913.8 (−1971.7, −1856.0)	−2413.3 (−2900.8, −1925.8)	−1186.4 (−1601.6, −771.3)	−1868.6 (−2410.1, −1327)	−974.4 (−1447.6, −501.3)
***Anxiety***						
**Employment**	*N* = 393,141	*N* = 390,076	*N* = 16,683	*N* = 15,689	*N* = 16,387	*N* = 15,907
Unemployed	1.30 (1.28–1.32)	1.22 (1.20–1.24)	1.28 (1.23–1.34)	1.15 (1.10–1.27)	1.16 (1.11–1.22)	1.12 (1.07–1.17)
**Income**	*N* = 393,086	*N* = 390,009	*N* = 16,673	*N* = 15,678	*N* = 16,380	*N* = 15,899
Disposable income (top-coded)	−1268.0 (−1371.5, −1164.5)	−904.3 (−991.1, −817.5)	−1216.5 (−1529.2, −903.9)	−555.8 (−799.5, −312.2)	−797.6 (−1136.9, −458.3)	−509.2 (−771.4, −246.9)
Earnings^**^	−2994.1 (−3069.7, −2919.5)	−1940.1 (−2012.8, −1867.5)	−2648.8 (−3199.1, −2098.5)	−1371.0 (−1839.1, −903.0)	−1839.3 (−2466.7, −1212)	−994.5 (−1521.0, −467.9)
***Depression***						
**Employment**	*N* = 320,877	*N* = 318,586	*N* = 13,051	*N* = 12,218	*N* = 12,908	*N* = 12,486
Unemployed	1.35 (1.33–1.38)	1.25 (1.23–1.27)	1.30 (1.23–1.37)	1.15 (1.09–1.21)	1.23 (1.16–1.30)	1.13 (1.07–1.19)
**Income**	*N* = 320,873	*N* = 318,580	*N* = 13,047	*N* = 12,214	*N* = 12,905	*N* = 12,483
Disposable income (top-coded)	−1425.5 (−1540.3, −1310.6)	−1024.4 (−1120.7, −928.0)	−1184.0 (−1542.0, −826.1)	−436.3 (−720.2, −152.3)	−923.9 (−1317.4, −530.3)	−486.9 (−793.0, −180.8)
Earnings^**^	−3390.7 (−3622.6, −3158.8)	−2302.0 (−2502.3, −2101.7)	−2660.8 (−3299, −2022.7)	−1268.6 (−1819.9, −717.4)	−2253.3 (−2985.2, −1521.3)	−1171.7 (−1773.5, −569.8)
***Schizophrenia***						
**Employment**	*N* = 100,970	*N* = 100,084	*N* = 4,081	*N* = 3,869	*N* = 4,112	*N* = 3,982
Unemployed	1.53 (1.49–1.58)	1.43 (1.39–1.47)	1.36 (1.23–1.50)	1.31 (1.18–1.44)	1.26 (1.15–1.39)	1.22 (1.11–1.35)
**Income**	*N* = 100,964	*N* = 100,073	*N* = 4,081	*N* = 3,869	*N* = 4,112	*N* = 3,982
Disposable income (top-coded)	−2004.5 (−2215, −1794)	−1512.8 (−1693.1, −1332.6)	−1247.2 (−1970.9, −586.5)	−945.8 (−1468.9, −422.7)	−781.4 (−1490.1, −72.7)	−354.8 (−917.7, 208.1)
Earnings^**^	−4039.4 (−4186.6, −3892.1)	−3017.2 (−3160.6, −2873.9)	−3337.1 (−4555.5, −2118.7)	−2289.2 (−3402.3, −1176.1)	−2541.5 (−3814.9, −1268.0)	−1486.0 (−2593.9, −378.1)
***Bipolar disorder***						
**Employment**	*N* = 30,471	*N* = 30,228	*N* = 1,185	*N* = 1,100	*N* = 1,451	*N* = 1,401
Unemployed	1.49 (1.41–1.58)	1.31 (1.24–1.38)	1.34 (1.10–1.63)	1.36 (1.12–1.66)	1.31 (1.11–1.56)	1.10 (0.93–1.31)
**Income**	*N* = 30,470	*N* = 30,227	*N* = 1,185	*N* = 1,100	*N* = 1,451	*N* = 1,401
Disposable income (top-coded)	−1853.3 (−2248, −1458.6)	−1209.6 (−1546.5, −872.8)	−552.0 (−1847.7, 743.8)	−68.0 (−1137.2, 1001.1)	−1329.2 (−2552.2, −106.2)	−379.6 (−1341.2, 582.0)
Earnings^**^	−3917.8 (−4807.9, −3027.7)	−2420.4 (−3178.5, −1662.3)	−2185.2 (−4675.7, 305.4)	−1461.0 (−3486.6, 664.7)	−3673.1 (−5838.2, −1507.9)	−1625.6 (−3397.0, −145.7)

The reference group was the siblings of unaffected probands

Adjusted: sex, age, residential region at baseline, any MPD diagnosis in the siblings before baseline, paternal and maternal highest education level at baseline, paternal and maternal history of any mental disorders before baseline, paternal and maternal age at birth of siblings of the affected and unaffected probands and the square of paternal and maternal age

The estimates for employment are ORs and 95% CI. The estimates for income are coefficient and 95%CI

^**^Additional analysis

### Associations across different subgroups

Results from the analyses stratified by the sex of siblings and age of probands at baseline are reported in Tables 4 and 5, S5–S8, and S9–S12. Overall, the patterns were relatively mixed across strata. For instance, the associations of having a sibling with an MPD diagnosis with the SES outcomes measured at the end of follow-up were larger among siblings whose affected probands were diagnosed at a younger age (Table 4, S5–S8). However, no similar pattern was consistently observed in the GEE models including the repeated measurements of income and employment each year since baseline (Table 5, S9–S12). The income gaps were consistently more significant among the affected probands’ male siblings than their female counterparts. For example, the adjusted difference in disposable income between male full siblings of the probands with and without depression was 1410.2 EUR. In contrast, it was 657.7 EUR for female full siblings of the probands with and without depression (Table S11).

**Table 4:** Associations between having an affected proband with *any MPDs* and subsequent SES at the end of follow-up across different subgroups (End-point analysis)

	Full siblings		Maternal half-siblings		Paternal half-siblings	
	Crude	Adjusted	Crude	Adjusted	Crude	Adjusted
**Any MPDs**						
**Education**	*N* = 386,360	*N* = 383,173	*N* = 13,740	*N* = 12,975	*N* = 13,047	*N* = 12,684
***Not having a higher education degree***						
*Sex*						
Men	0.70 (0.67–0.72)	1.24 (1.19–1.29)	0.72 (0.61–0.84)	1.17 (1.00-1.38)	0.75 (0.65–0.87)	1.24 (1.19–1.29)
Women	0.66 (0.64–0.68)	1.31 (1.27–1.36)	0.75 (0.66–0.85)	1.22 (1.07–1.39)	0.75 (0.66–0.85)	1.31 (1.27–1.36)
*Age of probands at baseline, years*						
5–14	0.53 (0.48–0.59)	1.52 (1.36–1.70)	0.52 (0.38–0.71)	1.67 (1.19–2.35)	0.58 (0.43–0.79)	1.52 (1.36–1.70)
15–19	0.70 (0.67–0.73)	1.25 (1.20–1.31)	0.78 (0.66–0.91)	1.08 (0.91–1.27)	0.80 (0.69–0.94)	1.25 (1.20–1.31)
20–25	0.69 (0.67–0.72)	1.27 (1.22–1.32)	0.77 (0.67–0.89)	1.19 (1.03–1.38)	0.77 (0.68–0.88)	1.27 (1.22–1.32)
**Employment**	*N* = 354,626	*N* = 351,766	*N* = 12,655	*N* = 11,918	*N* = 12,105	*N* = 11,773
***Unemployed***						
*Sex*						
Men	1.70 (1.63–1.77)	1.41 (1.36–1.47)	1.47 (1.30–1.67)	1.35 (1.18–1.54)	1.31 (1.15–1.50)	1.21 (1.05–1.39)
Women	1.52 (1.46–1.58)	1.30 (1.24–1.35)	1.38 (1.21–1.57)	1.26 (1.09–1.45)	1.35 (1.18–1.55)	1.24 (1.08–1.43)
*Age of probands at baseline, years*						
5–14	1.85 (1.65–2.07)	1.62 (1.44–1.82)	1.09 (0.83–1.44)	0.98 (0.73–1.33)	1.52 (1.12–2.05)	1.40 (1.03–1.91)
15–19	1.55 (1.48–1.63)	1.36 (1.29–1.42)	1.50 (1.29–1.73)	1.37 (1.18–1.59)	1.42 (1.21–1.67)	1.33 (1.13–1.56)
20–25	1.62 (1.56–1.69)	1.32 (1.27–1.37)	1.46 (1.28–1.66)	1.34 (1.16–1.53)	1.21 (1.06–1.39)	1.12 (0.97–1.29)
**Income (median**,** EUR)**	*N* = 354,506	*N* = 351,762	*N* = 12,635	*N* = 11,918	*N* = 12,101	*N* = 11,773
***Disposable income (top-coded)***						
*Sex*						
Men	−2448,7 (−2658.2, −2239.2)	−1678.5 (−1890.7, −1466.3)	−1489.1 (−2178.2, −799.9)	−1215.8 (−1902.5, −529.1)	−1438.4 (−2128.1, −748.8)	−986.4 (−1735.0, −237.8)
Women	−1359.6 (−1524.0, −1195.2)	−946.3 (−1114.7, −778.0)	−510.0 (−1061.1, 41.1)	−735.2 (−1302.0, −168.4)	−708.1 (−1288.5, −127.7)	−740.0 (−1303.3, −176.6)
*Age of probands at baseline, years*						
5–14	−2618.6 (−3123.8, −2113.3)	−1787.1 (−2338.8, −1235.3)	−864.1 (−2153.7, 425.4)	−229.1 (−1371.7, 913.5)	−1398.3 (−2992.4, 195.7)	−1424.5 (−2846.3, −2.8)
15–19	−1740.2 (−1966.6, −1513.7)	−1318.8 (−1534.3, −1103.2)	−969.9 (−1687.1, −252.6)	−1122.6 (−1762.5, −482.7)	−1067.7 (−1775.8, −359.5)	−846.2 (−1611.3, −81.2)
20–25	−2029.9 (−2202.6, −1857.1)	−1191.6 (−1369.7, −1013.4)	−1021.4 (−1626.2, −416.6)	−708.2 (−1353.3, −63.1)	−925.3 (−1507.2, −343.3)	−688.3 (−1305.9, −70.7)

The reference group was the siblings of unaffected probands

Adjusted: sex, age, residential region at baseline, any MPD diagnosis in the siblings before baseline, paternal and maternal highest education level at baseline, paternal and maternal history of any mental disorders before baseline, paternal and maternal age at birth of siblings of the affected and unaffected probands and the square of paternal and maternal age

The estimates for education achievement and employment are ORs and 95% CI

^*^ The estimates for income are coefficient and 95%CI

**Table 5:** Associations between having an affected proband with any MPDs and subsequent SES over the observation period across different subgroups (Longitudinal analysis)

	Full siblings		Maternal half-siblings		Paternal half-siblings	
	Crude	Adjusted	Crude	Adjusted	Crude	Adjusted
**Any MPDs**						
**Employment**	*N* = 593,536	*N* = 588,646	*N* = 20,350	*N* = 19,171	*N* = 19,457	*N* = 18,908
***Unemployed***						
*Sex*						
Men	1.36 (1.34–1.39)	1.25 (1.23–1.27)	1.35 (1.27–1.43)	1.21 (1.14–1.29)	1.16 (1.09–1.23)	1.11 (1.04–1.18)
Women	1.27 (1.25–1.29)	1.18 (1.17–1.20)	1.19 (1.13–1.26)	1.12 (1.06–1.19)	1.23 (1.16–1.31)	1.16 (1.10–1.23)
*Age of probands at baseline, years*						
5–14	1.32 (1.27–1.37)	1.26 (1.21–1.31)	1.17 (1.05–1.31)	1.09 (0.98–1.21)	1.34 (1.19–1.51)	1.28 (1.14–1.43)
15–19	1.24 (1.22–1.26)	1.18 (1.16–1.20)	1.32 (1.24–1.42)	1.18 (1.11–1.26)	1.15 (1.08–1.24)	1.13 (1.05–1.21)
20–25	1.39 (1.27–1.41)	1.24 (1.22–1.26)	1.25 (1.18–1.33)	1.17 (1.10–1.24)	1.19 (1.12–1.26)	1.12 (1.05–1.19)
**Income**	*N* = 593, 464	*N* = 588, 574	*N* = 20,346	*N* = 19,164	*N* = 19,451	*N* = 18,901
***Disposable income (top-coded)***						
*Sex*						
Men	−1868.9 (−2002.8, −1734.9)	−1295.4 (−1409.0, −1181.7)	−1634.8 (−2076.8, −1192.9)	−714.5 (−1064.5, −364.5)	−971.8 (−1450.1, −493.5)	−661.6 (−1040.2, −283.0)
Women	−784.4 (−884.3, −684.6)	−602.6 (−686.5, −518.6)	−524.3 (−868.1, −180.4)	−228.4 (−479.8, 23.0)	−677.1 (−1057.9, −296.2)	−347.1 (−635.0, −59.1)
*Age of probands at baseline, years*						
5–14	−1678.4 (−1981.6, −1375.3)	−1334.4 (−1595.3, −1073.5)	−887.2 (−1602.4, −172.1)	−169.6 (−735.4, 396.1)	−1676.8 (−2503.5, −850.1)	−1170.1 (−1842.3, −497.9)
15–19	−975.0 (−1106.0, −844.1)	−742.4 (−851.0, −633.8)	−1272.1 (−1728.2, −816.0)	−491.7 (−834.6, −148.9)	−794.2 (−1283.2, −305.1)	−620.9 (−996.9, −245.0)
20–25	−1640.9 (−1755.6, −1526.2)	−1051.0 (−1150.3, −951.8)	−993.7 (−1390.7, −596.8)	−496.3 (−807.4, −185.1)	−677.5 (−1113.4, −241.5)	−281.7 (−623.3, −60.0)
**Earnings** ^**^						
*Sex*						
Men	−4017.8 (−4279.7, −3755.8)	−2706.8 (−2930.8, −2482.9)	−3155.3 (−3961.0, −2349.5)	−1463.9 (−2160.8, −767.0)	−1808.8 (−2688.6, −929.0)	−901.6 (−1686.0, −117.2)
Women	−2160.3 (−2224.9, −2095.8)	−1364.4 (−1427.4, −1301.3)	−1569.1 (−2104.1, −1034.0)	−892.6 (−1348.1, −437.1)	−1753.1 (−2348.3, −1157.9)	−1065.9 (−1584.2, −547.7)
*Age of probands at baseline, years*						
5–14	−3346.8 (−3935.8, −2757.7)	−2430.1 (−2942.0, −1918.3)	−1666.3 (−2854.7, −477.9)	−514.1 (−1562.7, 534.6)	−3148.6 (−4496.2, −1801.0)	−2336.0 (−3561.4, −1110.6)
15–19	−2375.3 (−2470.1, −2280.4)	−1558.1 (−1650.1, −1466.2)	−2555.7 (−3343.0, −1768.4)	−1253.2 (−1931.1, −575.3)	−1954.6 (−2781.2, −1128.1)	−1464.3 (−2161.0, −767.6)
20–25	−3604.8 (−3688.2, −3521.4)	−2085.9 (−2165.6, −2006.5)	−2491.2 (−3202.3, −1780.1)	−1292.7 (−1894.4, −691.0)	−1503.7 (−2306.6, −700.8)	−411.6 (−1128.6, 305.4)

The reference group was the siblings of unaffected probands

Adjusted: sex, age, residential region at baseline, any MPD diagnosis in the siblings before baseline, paternal and maternal highest education level at baseline, paternal and maternal history of any mental disorders before baseline, paternal and maternal age at birth of siblings of the affected and unaffected probands and the square of paternal and maternal age

The estimates for employment are ORs and 95% CI

^*^The estimates for income are mean and 95%CI. ^**^Additional analysis

After excluding social benefits from the income measure, we observed that the earnings gaps between siblings of affected and unaffected probands nearly doubled across all MPDs and each specific mental disorder of interest (Tables 3 and 5 & S9-S12). For instance, compared to the maternal half-siblings of the unaffected probands, maternal half-siblings of the affected probands with anxiety disorders had 555.8 EUR lower disposable income (95% CI −799.5, −312.2) over the whole observation period, whereas the corresponding difference in earnings after excluding social security benefits was 1371 EUR (95% CI −1839.1, −903.0) (Table 3).

## Discussion

Using Finnish nationwide registry data, we showed that siblings of individuals with diagnosed MPDs in childhood and early adulthood were consistently at a greater risk of not achieving higher education, being unemployed, and had lower annual disposable income than siblings of individuals without MPDs. Stronger associations were observed among full siblings compared to maternal and paternal half-siblings. These findings suggest that having a sibling with any MPD diagnosed between the ages of 5 and 25 years is associated with unfavorable SES outcomes.

Our findings are in accordance with previous findings on the burden of caregiving associated with mental disorders among family members, including siblings [20, 31]. The results also align with a recent Danish cohort study, which reported that full siblings of patients with bipolar disorder were less likely to attain higher education achievement (>13 years of education), were more likely unemployed, and had lower income [22]. Similarly, a case-control study from the Netherlands observed lower educational achievement among full siblings of patients with schizophrenia [24]. However, these studies are not directly comparable to ours due to differences between study designs. First, while the Danish and Dutch studies compared healthy siblings of patients with bipolar disorder and schizophrenia to general population controls matched with the healthy siblings, we compared siblings of patients with diagnosed MPDs to siblings of individuals without any diagnosed MPDs, which can be interpreted as a “head-to-head” comparison. Second, our patient samples were younger on average. For example, the median age at baseline of the affected probands with any MPDs in our study was 20 years, whereas it was 45 years in the Danish study and 30 years in the Dutch study. Thus, our study provided a broader perspective on how having a sibling with any major mental disorder is associated with individuals’ SES outcomes in the long run.

The estimates were generally attenuated after adjusting for potential confounders. An exception to this pattern was the estimates for not having a higher education, which reversed direction after the adjustment. The largest associations with income and unemployment status over the observation period were consistently observed among full siblings of the affected probands with schizophrenia spectrum disorders [32]. The severe and chronic symptoms of schizophrenia spectrum disorders in young patients can be expected to influence negatively the co-siblings’ wellbeing in a shared environment [18, 33, 34]. Schizophrenia spectrum disorders, in particular, have been linked to poor performance at school and work in young individual [9], and our findings suggested that the disabling nature of these disorders was also significantly associated with academic and work performance in siblings. Meanwhile, it has been reported worldwide that depression and anxiety are two of the most prevalent and burdensome mental disorders among young people [35]. In our study, we found that having a full sibling with depression or anxiety disorder, as well as bipolar disorder, was likewise significantly associated with individual subsequent SES. The burden associated with these disorders in families with multiple children warrants greater attention in future research.

Examining the SES of this large cohort of siblings of affected probands with any MPD enhances our understanding of the burden and societal impact of these disorders, as well as relationship with productivity impairment in close family members. By excluding social benefits, the earning gaps between siblings of the affected and unaffected probands nearly doubled across any MPD after we redefined disposable income. This finding shows that the Nordic social welfare system and income transfers, to some extent, mitigate disposable income differences even though we still observed the gaps between the siblings of the affected and unaffected probands. These differences are likely more pronounced in settings where a universal social safety net and social income transfers typical of welfare states are not implemented.

### Strengths and limitations

To our knowledge, the present study is the most comprehensive analysis of the associations between having an affected proband and subsequent SES, focusing on diagnoses occurring in childhood or early adulthood. By utilizing nationwide register-based data, we mitigated misclassifying MPDs and demographic covariates. Psychiatric diagnoses reported in the FCR have been reported to have a good validity [36]. We also adjusted for any MPD diagnosis before baseline in the siblings to address the possible concern that the observed associations may be explained by the strong relationship between MPDs and SES in the same individuals (i.e., siblings of affected probands). Consistently, the observed associations further support the greater risks of adverse SES outcomes among siblings of individuals with any MPD [29]. In addition, it has been well established that schizophrenia considerably impacts the quality of life of patients’ siblings [34]. At the same time, no previous studies investigated other common mental disorders, which we were included in this study for the first time.

We acknowledge some limitations. First, in analyses examining sibling’s SES at the end of follow-up, selection bias is likely to be introduced due to missing SES records at the end of follow-up. This concern was yet mitigated in the GEE models using repeated measurements of income and employment each year since baseline, improving the robustness of our findings. Second, the MPDs among the affected probands were identified based on the secondary health care registers. While these registers likely include diagnoses of severe MPDs, siblings of probands whose MPDs were untreated or treated solely in the primary health care were misclassified as having an unaffected proband in our analyses. Third, we cannot rule out the possibility of unmeasured or residual confounding, which could bias the observed associations, and our findings do not have a causal interpretation. Fourth, although we used comprehensive nationwide registry data, the number of individuals diagnosed with bipolar disorder in childhood and early adulthood was relatively low, resulting in less precise estimates, especially for maternal half-siblings of affected probands with bipolar disorders, which is likely partially explained by the complex nature of bipolar disorders and the challenges of differential diagnoses among children and adolescents [37]. Finally, the socioeconomic context in Finland differs from many other countries, with numerous programs and benefits available to support individuals and families with mental disorders. Consequently, our results may not be generalizable to high-income countries with different social welfare systems.

## Conclusion

Having a sibling with a major psychiatric disorder diagnosed in childhood or early adulthood was consistently associated with lower subsequent educational achievement, unemployment, and reduced income. Our findings suggest the potential relevance of early support to siblings of young patients with MPDs.

## Supplementary Information

Below is the link to the electronic supplementary material.

Supplementary Material 1

## Data Availability

No datasets were generated or analysed during the current study.

## Abbreviations

MPD: Major psychiatric disorders
SES: Socioeconomic status
GEE: Generalized Estimating Equations
aOR: Adjusted odds ratio
95%CI: 95% confidence interval
ICD: International Statistical Classification of Diseases
FCR: Finnish Care Register
