# Supplementary material for: Four major psychiatric disorders in childhood and early adulthood and siblings’ subsequent socioeconomic status: a nationwide register study
Source: Soc Psychiatry Psychiatr Epidemiol. 2025 Sep 27;61(4):649–61. doi: 10.1007/s00127-025-02997-y (PMC13021809; doi:10.1007/s00127-025-02997-y)
Supplement: Supplementary file 1 — Supplementary Material 1 [file 127_2025_2997_MOESM1_ESM.docx]

**Supplementary**

**Social Psychiatry and Psychiatric Epidemiology**

**Title: Four Major Psychiatric Disorders in Childhood and Early Adulthood and Siblings' Subsequent Socioeconomic Status: A nationwide register study**

Wen Yang^1^, Kaisla Komulainen^1^, Ripsa Niemi^1^, Mai Gutvilig^1^, Petri Böckerman^3,4,5^, Marko Elovainio^1,2^ & Christian Hakulinen^1,2^

^1^Department of Psychology, Faculty of Medicine, University of Helsinki, Helsinki, Finland

^2^Finnish Institute for Health and Welfare, Helsinki, Finland

^3^School of Business and Economics, University of Jyväskylä, Jyväskylä, Finland

^4^Labour Institute for Economic Research LABORE, Helsinki, Finland

^5^IZA Institute of Labor Economics, Germany

**Corresponding authors:** Wen Yang, University of Helsinki, P.O. Box 21, 00014, Helsinki, Finland. Email: wen.yang@helsinki.fi & Christian Hakulinen, University of Helsinki, P.O. Box 21, 00014, Helsinki, Finland. Tel: +358 50 448 2041 Email: christian.hakulinen@helsinki.fi

**Table of Contents**

[Detailed description of used registers 3](#_Toc205547209)

[Table S1. Descriptive characteristics in siblings of the affected and unaffected probands with diagnosed schizophrenia spectrum disorders 4](#_Toc205547210)

[Table S2. Descriptive characteristics in siblings of the affected and unaffected probands with diagnosed bipolar disorders 6](#_Toc205547211)

[Table S3. Descriptive characteristics in siblings of the affected and unaffected probands with diagnosed depressive disorders 8](#_Toc205547212)

[Table S4. Descriptive characteristics in siblings of the affected and unaffected probands with diagnosed anxiety disorders 10](#_Toc205547213)

[Table S5. Associations between having an affected proband with *schizophrenia spectrum disorders* and subsequent SES at the end of follow-up across different subgroups (End-point analysis). 12](#_Toc205547214)

[Table S6. Associations between having an affected proband with *bipolar disorder* and subsequent SES at the end of follow-up across different subgroups (End-point analysis). 14](#_Toc205547215)

[Table S7. Associations between having an affected proband with *depressive disorders* and subsequent SES at the end of follow-up across different subgroups (End-point analysis). 16](#_Toc205547216)

[Table S8. Associations between having an affected proband with *anxiety disorders* and subsequent SES at the end of follow-up across different subgroups (End point analysis). 18](#_Toc205547217)

[Table S9. Associations between having an affected proband with schizophrenia spectrum disorders and subsequent SES over the observation period across different subgroups (Longitudinal analysis). 20](#_Toc205547218)

[Table S10. Associations between having an affected proband with bipolar disorders and subsequent SES over the observation period across different subgroups (Longitudinal analysis). 22](#_Toc205547219)

[Table S11. Associations between having an affected proband with depressive disorders and subsequent SES over the observation period across different subgroups (Longitudinal analysis). 24](#_Toc205547220)

[Table S12. Associations between having an affected proband with anxiety disorders and subsequent SES over the observation period across different subgroups (Longitudinal analysis). 26](#_Toc205547221)

[Figure S1. Study sample derivation, full sibling cohorts. 28](#_Toc205547222)

[Figure S2. Study sample derivation, maternal half sibling cohorts. 29](#_Toc205547223)

[Figure S3. Study sample derivation, paternal half sibling cohorts. 30](#_Toc205547224)

# Detailed description of used registers

- FOLK modules of Statistics Finland: FOLK modules cover complete demographic and family data. They record on the periods of employment, income and annual earnings from the state-run pension and tax registers that cover all legal employment contracts. Complete data are available from 1987 onwards.
- Finnish Care Register (FCR): contains comprehensive information on secondary health care including visits to hospitals, including emergency department, inpatient, and outpatient records. Both public and private institutions are included. Data are available from 1970 onwards. The FCR used the Finnish versions of the International Classification of Diseases (ICD-8: 1970-1986; ICD-9:1987-1995; ICD-10: 1996 onwards) to record diagnosis.

# Table S1. Descriptive characteristics in siblings of the affected and unaffected probands with diagnosed schizophrenia spectrum disorders

|  | **Full siblings** | | **Maternal half-siblings** | | **Paternal half-siblings** | |
| --- | --- | --- | --- | --- | --- | --- |
|  | Unaffected | Affected | Unaffected | Affected | Unaffected | Affected |
| **Total (N)** | 91,015 | 10,132 | 3,117 | 968 | 3,146 | 976 |
| **Sex, n (%)** |  |  |  |  |  |  |
| Men | 46,146 (50.7%) | 5,126 (50.6%) | 1,574 (50.5%) | 478 (49.4%) | 1,585 (50.4%) | 467 (47.8%) |
| Women | 44,869 (49.3%) | 5,006 (49.4%) | 1,543 (49.5%) | 490 (50.6%) | 1,561 (49.6%) | 509 (52.2%) |
| **Age at baseline, years^*^** | 20.0 (16.0-25.0) | 21.0 (17.0-25.0) | 18.0 (12.0-27.0) | 19.0 (13.0-27.0) | 20.0 (13.0-27.0) | 19.0 (13.0-27.0) |
| **Migration status, n (%)** |  |  |  |  |  |  |
| Finnish born | 90,959 (99.9%) | 10,126 (99.9%) | 3,117 (100.0%) | 968 (100.0%) | 3,141 (99.8%) | 974 (99.8%) |
| Second-generation migrant | 56 ( 0.1%) | 6 ( 0.1%) | NA^*^ | NA | 5 ( 0.2%) | NA |
| **Any MPD diagnosis before baseline, n (%)** | 3,052 (3.4%) | 1,009 (10.0%) | 182 (5.8%) | 88 (9.1%) | 163 (5.2%) | 89 (9.1%) |
| **Residential Region at baseline, n (%)** |  |  |  |  |  |  |
| Helsinki Central Area | 27,591 (30.3%) | 3,278 (32.4%) | 1,000 (32.1%) | 326 (33.7%) | 1,235 (39.3%) | 363 (37.2%) |
| Southern Finland | 16,966 (18.6%) | 1,840 (18.2%) | 859 (27.6%) | 241 (24.9%) | 718 (22.8%) | 223 (22.8%) |
| Western Finland | 18,800 (20.7%) | 2,016 (19.9%) | 577 (18.5%) | 200 (20.7%) | 641 (20.4%) | 180 (18.4%) |
| Northern and Eastern Finland | 27,581 (30.3%) | 2,996 (29.6%) | 670 (21.5%) | 196 (20.2%) | 544 (17.3%) | 204 (20.9%) |
| Missing | 77 (0.1%) | NA | 11 (0.4%) | 5 (0.5%) | 8 (0.3%) | 6 (0.6%) |
|  |  |  |  |  |  |  |
| ***Characteristics of probands*** |  |  |  |  |  |  |
| **Sex, n (%)** |  |  |  |  |  |  |
| Men | 53,914 (59.2%) | 5,906 (58.3%) | 1,926 (61.8%) | 585 (60.4%) | 1,907 (60.6%) | 587 (60.1%) |
| Women | 37,101 (40.8%) | 4,226 (41.7%) | 1,191 (38.2%) | 383 (39.6%) | 1,239 (39.4%) | 389 (39.9%) |
| **Age of probands at baseline (years), n (%)** |  |  |  |  |  |  |
| 5-14 | 2,959 (3.2%) | 308 (3.1%) | 133 (4.2%) | 43 (4.4%) | 117 (3.7%) | 35 (3.6%) |
| 15-19 | 30,295 (33.3%) | 3,291 (32.5%) | 1,076 (34.5%) | 342 (35.3%) | 1,223 (38.9%) | 355 (36.4%) |
| 20-25 | 57,761 (63.5%) | 6,533 (64.5%) | 1,908 (61.2%) | 583 (60.2%) | 1,806 (57.4%) | 586 (60.0%) |
|  |  |  |  |  |  |  |
| ***Characteristics of parents*** |  |  |  |  |  |  |
| **Paternal age at birth of siblings, years^*^** | 29.0 (26.0-33.0) | 29.0 (26.0-34.0) | 27.0 (24.0-33.0) | 27.0 (24.0-33.0) | 28.0 (24.0-33.0) | 29.0 (24.0-34.0) |
| **Maternal age at birth of siblings, years^*^** | 27.0 (24.0-31.0) | 27.0 (24.0-32.0) | 25.0 (21.0-30.0) | 25.0 (21.0-31.0) | 25.0 (22.0-30.0) | 25.0 (21.0-30.0) |
| **Paternal highest education level at baseline, n (%)** |  |  |  |  |  |  |
| Lower secondary or less | 24,659 (27.1%) | 2,794 (27.6%) | 1,033 (33.1%) | 317 (32.7%) | 1,097 (34.9%) | 346 (35.5%) |
| Upper secondary | 32,033 (35.2%) | 3,396 (33.5%) | 1,151 (36.9%) | 375 (38.7%) | 1,126 (35.8%) | 328 (33.6%) |
| Post-secondary or tertiary | 29,725 (32.7%) | 3,230 (31.9%) | 456 (14.6%) | 123 (12.7%) | 639 (20.3%) | 199 (20.4%) |
| Missing | 4,598 ( 5.1%) | 712 ( 7.0%) | 477 (15.3%) | 153 (15.8%) | 284 ( 9.0%) | 103 (10.6%) |
| **Maternal highest education level at baseline, n (%)** |  |  |  |  |  |  |
| Lower secondary or less | 20,734 (22.8%) | 2,471 (24.4%) | 1,080 (34.6%) | 389 (40.2%) | 948 (30.1%) | 312 (32.0%) |
| Upper secondary | 37,896 (41.6%) | 4,105 (40.5%) | 1,366 (43.8%) | 399 (41.2%) | 1,311 (41.7%) | 380 (38.9%) |
| Post-secondary or tertiary | 30,373 (33.4%) | 3,280 (32.4%) | 566 (18.2%) | 132 (13.6%) | 732 (23.3%) | 225 (23.1%) |
| Missing | 2,012 ( 2.2%) | 276 ( 2.7%) | 105 ( 3.4%) | 48 ( 5.0%) | 155 ( 4.9%) | 59 ( 6.0%) |
| **Paternal history of mental disorder prior to the baseline, yes, n (%)** | 7,242 ( 8.0%) | 1,759 (17.4%) | 642 (20.6%) | 224 (23.1%) | 519 (16.5%) | 281 (28.8%) |
| **Maternal history of mental disorder prior to the baseline, yes, n (%)** | 6,097 ( 6.7%) | 1,699 (16.8%) | 499 (16.0%) | 298 (30.8%) | 437 (13.9%) | 148 (15.2%) |

**^*^**Median and IQR

NA: data unavailable due to very few observations (<3).

Differences in baseline characteristics (i.e., from the date of the first diagnosis of any MPDs) were tested using the χ^2^ test for categorical variables and the Mann-Whitney U test for continuous variables, *P*<0.001 for all.

# Table S2. Descriptive characteristics in siblings of the affected and unaffected probands with diagnosed bipolar disorders

|  | **Full siblings** | | **Maternal half-siblings** | | **Paternal half-siblings** | |
| --- | --- | --- | --- | --- | --- | --- |
|  | Unaffected | Affected | Unaffected | Affected | Unaffected | Affected |
| **Total (N)** | 27,589 | 2,948 | 910 | 279 | 1,101 | 350 |
| **Sex, n (%)** |  |  |  |  |  |  |
| Men | 14,128 (51.2%) | 1,458 (49.5%) | 464 (51.0%) | 130 (46.6%) | 568 (51.6%) | 179 (51.1%) |
| Women | 13,461 (48.8%) | 1,490 (50.5%) | 446 (49.0%) | 149 (53.4%) | 533 (48.4%) | 171 (48.9%) |
| **Age at baseline, years^*^** | 22.0 (18.0-26.0) | 22.0 (18.0-26.0) | 26.0 (16.0-31.0) | 25.0 (16.0-30.0) | 26.0 (17.0-30.0) | 25.0 (17.0-31.0) |
| **Migration status, n (%)** |  |  |  |  |  |  |
| Finnish born | 27,571 (99.9%) | 2,948 (100.0%) | 908 (99.8%) | 279 (100.0%) | 1,101 (100.0%) | 345 (98.6%) |
| Second-generation migrant | 18 ( 0.1%) | NA | NA | NA | NA | NA |
| **Any MPD diagnosis before baseline, n (%)** | 1,266 (4.6%) | 365 (12.4%) | 80 (8.8%) | 36 (12.9%) | 84 (7.6%) | 48 (13.7%) |
| **Residential Region at baseline, n (%)** |  |  |  |  |  |  |
| Helsinki Central Area | 8,976 (32.5%) | 1,000 (33.9%) | 266 (29.2%) | 98 (35.1%) | 429 (39.0%) | 135 (38.6%) |
| Southern Finland | 5,304 (19.2%) | 542 (18.4%) | 234 (25.7%) | 62 (22.2%) | 231 (21.0%) | 69 (19.7%) |
| Western Finland | 5,499 (19.9%) | 619 (21.0%) | 171 (18.8%) | 58 (20.8%) | 235 (21.3%) | 62 (17.7%) |
| Northern and Eastern Finland | 7,799 (28.3%) | 785 (26.6%) | 239 (26.3%) | 60 (21.5%) | 206 (18.7) | 84 (24.0%) |
| Missing | 11 (<0.1%) | NA | NA | NA | NA | NA |
|  |  |  |  |  |  |  |
| ***Characteristics of probands*** |  |  |  |  |  |  |
| **Sex, n (%)** |  |  |  |  |  |  |
| Men | 10,138 (36.7%) | 1,127 (38.2%) | 282 (31.0%) | 89 (31.9%) | 420 (38.1%) | 144 (41.1%) |
| Women | 17,451 (63.3%) | 1,821 (61.8%) | 628 (69.0%) | 190 (68.1%) | 681 (61.9%) | 206 (58.9%) |
| **Age of probands at baseline (years), n (%)** |  |  |  |  |  |  |
| 5-14 | 291 (1.1%) | NA | 12 ( 1.3%) | NA | NA | NA |
| 15-19 | 4,954 (18.0%) | 567 (19.2%) | 159 (17.5%) | NA | 195 (17.7%) | 67 (19.1%) |
| 20-25 | 22,344 (81.0%) | 2,345 (79.5%) | 739 (81.2%) | 231 (82.8%) | 906 (82.3%) | 283 (80.9%) |
|  |  |  |  |  |  |  |
| ***Characteristics of parents*** |  |  |  |  |  |  |
| **Paternal age at birth of siblings, years^*^** | 29.0 (26.0-33.0) | 29.0 (26.0-33.0) | 26.0 (23.0-30.0) | 26.0 (23.0-31.0) | 27.0 (23.0-32.0) | 27.0 (23.0-32.0) |
| **Maternal age at birth of siblings, years^*^** | 27.0 (24.0-31.0) | 27.0 (23.0-31.0) | 24.0 (20.0-29.0) | 24.0 (20.0-28.0) | 24.0 (21.0-29.0) | 24.0 (21.0-29.0) |
| **Paternal highest education level at baseline, n (%)** |  |  |  |  |  |  |
| Lower secondary or less | 6,877 (24.9%) | 685 (23.2%) | 269 (29.6%) | 72 (25.8%) | 346 (31.4%) | 125 (35.7%) |
| Upper secondary | 10,043 (36.4%) | 1,043 (35.4%) | 366 (40.2%) | 125 (44.8%) | 411 (37.3%) | 121 (34.6%) |
| Post-secondary or tertiary | 9,180 (33.3%) | 985 (33.4%) | 100 (11.0%) | 32 (11.5%) | 229 (20.8%) | 74 (21.1%) |
| Missing | 1,489 ( 5.4%) | 235 ( 8.0%) | 175 (19.2%) | 50 (17.9%) | 115 (10.4%) | 30 ( 8.6%) |
| **Maternal highest education level at baseline, n (%)** |  |  |  |  |  |  |
| Lower secondary or less | 5,630 (20.4%) | 604 (20.5%) | 323 (35.5%) | 88 (31.5%) | 311 (28.2%) | 87 (24.9%) |
| Upper secondary | 11,642 (42.2%) | 1,172 (39.8%) | 438 (48.1%) | 130 (46.6%) | 450 (40.9%) | 151 (43.1%) |
| Post-secondary or tertiary | 9,719 (35.2%) | 1,084 (36.8%) | 131 (14.4%) | 48 (17.2%) | 265 (24.1%) | 93 (26.6%) |
| Missing | 598 ( 2.2%) | 88 ( 3.0%) | 18 ( 2.0%) | 13 ( 4.7%) | 75 ( 6.8%) | 19 ( 5.4%) |
| **Paternal history of mental disorder prior to the baseline, yes, n (%)** | 2,695 ( 9.8%) | 581 (19.7%) | 199 (21.9%) | 54 (19.4%) | 200 (18.2%) | 121 (34.6%) |
| **Maternal history of mental disorder prior to the baseline, yes, n (%)** | 2,335 ( 8.5%) | 565 (19.2%) | 160 (17.6%) | 97 (34.8%) | 167 (15.2%) | 72 (20.6%) |

**^*^**Median and IQR

NA: data unavailable due to very few observations (<3).

Differences in baseline characteristics (i.e., from the date of the first diagnosis of any MPDs) were tested using the χ^2^ test for categorical variables and the Mann-Whitney U test for continuous variables, *P*<0.001 for all.

# Table S3. Descriptive characteristics in siblings of the affected and unaffected probands with diagnosed depressive disorders

|  | **Full siblings** | | **Maternal half-siblings** | | **Paternal half-siblings** | |
| --- | --- | --- | --- | --- | --- | --- |
|  | Unaffected | Affected | Unaffected | Affected | Unaffected | Affected |
| **Total (N)** | 290,440 | 31,061 | 9,806 | 3,270 | 9,847 | 3,094 |
| **Sex, n (%)** |  |  |  |  |  |  |
| Men | 147,486 (50.8%) | 15,578 (50.2%) | 5,017 (51.2%) | 1,651 (50.5%) | 4,943 (50.2%) | 1,535 (49.6%) |
| Women | 142,954 (49.2%) | 15,483 (49.8%) | 4,789 (48.8%) | 1,619 (49.5%) | 4,904 (49.8%) | 1,559 (50.4%) |
| **Age at baseline, years^*^** | 20.0 (16.0-25.0) | 21.0 (16.0-25.0) | 20.0 (13.0-28.0) | 19.0 (13.0-27.0) | 21.0 (14.0-27.0) | 21.0 (13.0-28.0) |
| **Migration status, n (%)** |  |  |  |  |  |  |
| Finnish born | 290,331 (100.0%) | 31,055 (100.0%) | NA | NA | 9,843 (100.0%) | 3,094 (100.0%) |
| Second-generation migrant | 109 ( 0.0%) | 6 ( 0.0%) | NA | NA | 4 ( 0.0%) | NA |
| **Any MPD diagnosis before baseline, n (%)** | 9,806 (3.4%) | 3,337 (10.7%) | 568 (5.8%) | 353 (10.8%) | 554 (5.6%) | 258 (8.3%) |
| **Residential Region at baseline, n (%)** |  |  |  |  |  |  |
| Helsinki Central Area | 82,580 (28.4%) | 9,357 (30.1%) | 2,779 (28.3%) | 994 (30.4%) | 3,303 (33.5%) | 1,034 (33.4%) |
| Southern Finland | 57,855 (19.9%) | 6,263 (20.2%) | 2,563 (26.1%) | 812 (24.8%) | 2,324 (23.6%) | 679 (21.9%) |
| Western Finland | 66,931 (23.0%) | 6,995 (22.5%) | 2,272 (23.2%) | 721 (22.0%) | 2,221 (22.6%) | 697 (22.5%) |
| Northern and Eastern Finland | 82,953 (28.6%) | 8,430 (27.1%) | 2,139 (21.8%) | 728 (22.3%) | 1,969 (20.0%) | 668 (21.6%) |
| Missing | 121 (<0.1%) | 16 (0.1%) | 53 (0.5%) | 15 (0.5%) | 30 (0.3%) | 16 (0.5%) |
|  |  |  |  |  |  |  |
| ***Characteristics of probands*** |  |  |  |  |  |  |
| **Sex, n (%)** |  |  |  |  |  |  |
| Men | 115,212 (39.7%) | 12,272 (39.5%) | 4,680 (47.7%) | 1,540 (47.1%) | 4,515 (45.9%) | 1,452 (46.9%) |
| Women | 175,228 (60.3%) | 18,789 (60.5%) | 5,126 (52.3%) | 1,730 (52.9%) | 5,332 (54.1%) | 1,642 (53.1%) |
| **Age of probands at baseline (years), n (%)** |  |  |  |  |  |  |
| 5-14 | 12,168 (4.2%) | 1,224 (4.0%) | 787 (8.0%) | 260 (8.0%) | 717 (7.2%) | 243 (7.8%) |
| 15-19 | 106,954 (36.8%) | 11,199 (36.1%) | 3,728 (38.0%) | 1,227 (37.5%) | 3,437 (34.9%) | 1,080 (34.9%) |
| 20-25 | 171,318 (59.0%) | 18,638 (60.0%) | 5,291 (54.0%) | 1,783 (54.5%) | 5,693 (57.8%) | 1,771 (57.2%) |
|  |  |  |  |  |  |  |
| ***Characteristics of parents*** |  |  |  |  |  |  |
| **Paternal age at birth of siblings, years^*^** | 29.0 (26.0-33.0) | 29.0 (25.0-33.0) | 27.0 (23.0-32.0) | 27.0 (23.0-32.0) | 28.0 (24.0-33.0) | 28.0 (23.0-33.0) |
| **Maternal age at birth of siblings, years^*^** | 27.0 (24.0-31.0) | 26.0 (23.0-31.0) | 25.0 (21.0-29.0) | 25.0 (21.0-29.0) | 25.0 (21.0-29.0) | 24.0 (21.0-29.0) |
| **Paternal highest education level at baseline, n (%)** |  |  |  |  |  |  |
| Lower secondary or less | 76,603 (26.4%) | 8,646 (27.8%) | 3,197 (32.6%) | 1,048 (32.0%) | 3,290 (33.4%) | 1,063 (34.4%) |
| Upper secondary | 105,493 (36.3%) | 11,307 (36.4%) | 3,699 (37.7%) | 1,325 (40.5%) | 3,765 (38.2%) | 1,243 (40.2%) |
| Post-secondary or tertiary | 94,961 (32.7%) | 8,668 (27.9%) | 1,344 (13.7%) | 356 (10.9%) | 1,872 (19.0%) | 443 (14.3%) |
| Missing | 13,383 ( 4.6%) | 2,440 ( 7.9%) | 1,566 (16.0%) | 541 (16.5%) | 920 ( 9.3%) | 345 (11.2%) |
| **Maternal highest education level at baseline, n (%)** |  |  |  |  |  |  |
| Lower secondary or less | 62,395 (21.5%) | 7,601 (24.5%) | 3,462 (35.3%) | 1,257 (38.4%) | 2,874 (29.2%) | 1,001 (32.4%) |
| Upper secondary | 122,678 (42.2%) | 13,222 (42.6%) | 4,370 (44.6%) | 1,417 (43.3%) | 4,088 (41.5%) | 1,312 (42.4%) |
| Post-secondary or tertiary | 99,820 (34.4%) | 9,343 (30.1%) | 1,571 (16.0%) | 422 (12.9%) | 2,359 (24.0%) | 608 (19.7%) |
| Missing | 5,547 ( 1.9%) | 895 ( 2.9%) | 403 ( 4.1%) | 174 ( 5.3%) | 526 ( 5.3%) | 173 ( 5.6%) |
| **Paternal history of mental disorder prior to the baseline, yes, n (%)** | 24,109 ( 8.3%) | 5,146 (16.6%) | 2,011 (20.5%) | 796 (24.3%) | 1,719 (17.5%) | 875 (28.3%) |
| **Maternal history of mental disorder prior to the baseline, yes, n (%)** | 19,903 ( 6.9%) | 4,992 (16.1%) | 1,437 (14.7%) | 937 (28.7%) | 1,332 (13.5%) | 507 (16.4%) |

**^*^**Median and IQR

NA: data unavailable due to very few observations (<3).

Differences in baseline characteristics (i.e., from the date of the first diagnosis of any MPDs) were tested using the χ^2^ test for categorical variables and the Mann-Whitney U test for continuous variables, *P*<0.001 for all.

# Table S4. Descriptive characteristics in siblings of the affected and unaffected probands with diagnosed anxiety disorders

|  | **Full siblings** | | **Maternal half-siblings** | | **Paternal half-siblings** | |
| --- | --- | --- | --- | --- | --- | --- |
|  | Unaffected | Affected | Unaffected | Affected | Unaffected | Affected |
| **Total (N)** | 356,109 | 37,693 | 12,526 | 4,183 | 12,595 | 3,843 |
| **Sex, n (%)** |  |  |  |  |  |  |
| Men | 180,839 (50.8 %) | 18,904 (50.2 %) | 6,388 (51.0%) | 2,111 (50.5%) | 6,312 (50.1%) | 1,913 (49.8%) |
| Women | 175,270 (49.2%) | 18,789 (49.8%) | 6,138 (49.0%) | 2,072 (49.5%) | 6,283 (49.9%) | 1,930 (50.2%) |
| **Age at baseline, years^*^** | 20.0 (15.0–24.0) | 20.0 (16.0-24.0) | 18.0 (12.0-27.0) | 17.0 (11.0-26.0) | 18.0 (12.0-26.0) | 18.0 (12.0-26.0) |
| **Migration status, n (%)** |  |  |  |  |  |  |
| Finnish born | 355,956 (100.0%) | NA | NA | NA | 12,585 (99.9%) | 3,840 (99.9%) |
| Second-generation migrant | 153 ( 0.0%) | NA | NA | NA | 10 ( 0.1%) | 3 ( 0.1%) |
| **Any MPD diagnosis before baseline, n (%)** | 10,606 (3.0%) | 3,531 (9.4%) | 599 (4.8%) | 381 (9.1%) | 562 (4.5%) | 288 (7.5%) |
| **Residential Region at baseline, n (%)** |  |  |  |  |  |  |
| Helsinki Central Area | 106,066 (29.8%) | 11,691 (31.0%) | 3,510 (28.0%) | 1,337 (32.0%) | 4,225 (33.5%) | 1,335 (34.7%) |
| Southern Finland | 70,984 (19.9%) | 7,575 (20.1%) | 3,256 (26.0%) | 1,036 (24.8%) | 2,939 (23.3%) | 848 (22.1%) |
| Western Finland | 81,159 (22.8%) | 8,425 (22.4%) | 2,794 (22.3%) | 882 (21.1%) | 2,670 (21.2%) | 842 (21.9%) |
| Northern and Eastern Finland | 97,433 (27.4%) | 9,966 (26.4%) | 2,762 (22.1%) | 869 (20.8%) | 2,573 (20.4%) | 766 (19.9%) |
| Missing | 467 (0.1%) | 36 (0.1%) | 204 (1.6%) | 59 (1.4%) | 188 (1.5%) | 52 (1.4%) |
|  |  |  |  |  |  |  |
| ***Characteristics of probands*** |  |  |  |  |  |  |
| **Sex, n (%)** |  |  |  |  |  |  |
| Men | 191,652 (53.8%) | 19,892 (52.8%) | 7,383 (58.9%) | 2,439 (58.3%) | 7,335 (58.2%) | 2,227 (57.9%) |
| Women | 164,457 (46.2%) | 17,801 (47.2%) | 5,143 (41.1%) | 1,744 (41.7%) | 5,260 (41.8%) | 1,616 (42.1%) |
| **Age of probands at baseline (years), n (%)** |  |  |  |  |  |  |
| 5-14 | 25,800 (7.2%) | 2637 (7.0%) | 1681 (13.4%) | 594 (14.2%) | 1,681 (13.3%) | 506 (13.2%) |
| 15-19 | 142,212 (39.9%) | 14,639 (38.8%) | 4,984 (39.8%) | 1,620 (38.7%) | 4,898 (38.9%) | 1,486 (38.7%) |
| 20-25 | 188,097 (52.8%) | 20,417 (54.2%) | 5,861 (46.8%) | 1,969 (47.1%) | 6,016 (47.8%) | 1,851 (48.2%) |
|  |  |  |  |  |  |  |
| ***Characteristics of parents*** |  |  |  |  |  |  |
| **Paternal age at birth of siblings, years^*^** | 29.0 (26.0-33.0) | 29.0 (25.0-33.0) | 27.0 (23.0-32.0) | 27.0 (24.0-32.0) | 28.0 (24.0-33.0) | 28.0 (24.0-33.0) |
| **Maternal age at birth of siblings, years^*^** | 27.0 (24.0-31.0) | 26.0 (23.0-31.0) | 25.0 (21.0-30.0) | 25.0 (21.0-30.0) | 25.0 (21.0-29.0) | 24.0 (21.0-29.0) |
| **Paternal highest education level at baseline, n (%)** |  |  |  |  |  |  |
| Lower secondary or less | 95,554 (26.8%) | 11,169 (29.6%) | 4,113 (32.8%) | 1,435 (34.3%) | 4,205 (33.4%) | 1,418 (36.9%) |
| Upper secondary | 126,181 (35.4%) | 13,598 (36.1%) | 4,745 (37.9%) | 1,669 (39.9%) | 4,768 (37.9%) | 1,488 (38.7%) |
| Post-secondary or tertiary | 116,805 (32.8%) | 10,015 (26.6%) | 1,731 (13.8%) | 421 (10.1%) | 2,389 (19.0%) | 459 (11.9%) |
| Missing | 17,569 ( 4.9%) | 2,911 ( 7.7%) | 1,937 (15.5%) | 658 (15.7%) | 1,233 ( 9.8%) | 478 (12.4%) |
| **Maternal highest education level at baseline, n (%)** |  |  |  |  |  |  |
| Lower secondary or less | 79,402 (22.3%) | 10,218 (27.1%) | 4,471 (35.7%) | 1,728 (41.3%) | 3,697 (29.4%) | 1,309 (34.1%) |
| Upper secondary | 147,432 (41.4%) | 15,735 (41.7%) | 5,540 (44.2%) | 1,760 (42.1%) | 5,270 (41.8%) | 1,597 (41.6%) |
| Post-secondary or tertiary | 121,119 (34.0%) | 10,486 (27.8%) | 1,939 (15.5%) | 455 (10.9%) | 2,851 (22.6%) | 717 (18.7%) |
| Missing | 8,156 ( 2.3%) | 1,254 ( 3.3%) | 576 ( 4.6%) | 240 ( 5.7%) | 777 ( 6.2%) | 220 ( 5.7%) |
| **Paternal history of mental disorder prior to the baseline, yes, n (%)** | 27,961 ( 7.9%) | 5,670 (15.0%) | 2,476 (19.8%) | 898 (21.5%) | 2,085 (16.6%) | 977 (25.4%) |
| **Maternal history of mental disorder prior to the baseline, yes, n (%)** | 23,040 ( 6.5%) | 5,521 (14.6%) | 1,688 (13.5%) | 1,050 (25.1%) | 1,504 (11.9%) | 502 (13.1%) |

**^*^**Median and IQR

NA: data unavailable due to very few observations (<3).

Differences in baseline characteristics (i.e., from the date of the first diagnosis of any MPDs) were tested using the χ^2^ test for categorical variables and the Mann-Whitney U test for continuous variables, *P*<0.001 for all.

# Table S5. Associations between having an affected proband with *schizophrenia spectrum disorders* and subsequent SES at the end of follow-up across different subgroups (End-point analysis).

|  | **Full siblings** | | **Maternal half-siblings** | | **Paternal half-siblings** | |
| --- | --- | --- | --- | --- | --- | --- |
|  | Crude | Adjusted | Crude | Adjusted | Crude | Adjusted |
| **Education** | N=65,815 | N=65,228 | N=2,765 | N=2,626 | N=2,781 | N=2,687 |
| **Not having a higher education degree** | | | | | | |
| **Sex** | | | | | | |
| Men | 0.78 (0.72-0.85) | 1.21 (1.11-1.33) | 0.73 (0.51-1.04) | 1.18 (0.81-1.72) | 0.85 (0.61-1.19) | 1.21 (1.11-1.33) |
| Women | 0.71 (0.66-0.77) | 1.26 (1.16-1.38) | 0.87 (0.66-1.15) | 1.08 (0.80-1.46) | 0.89 (0.68-1.16) | 1.26 (1.16-1.38) |
| **Age of probands at baseline, years** | | | | | | |
| 5-14 | 0.60 (0.42-0.86) | 1.60 (1.09-2.35) | 1.17 (0.41-3.32) | 0.53 (0.15-1.92) | 1.04 (0.34-3.21) | 1.60 (1.09-2.35) |
| 15-19 | 0.83 (0.75-0.92) | 1.11 (1.00-1.25) | 0.96 (0.68-1.36) | 0.90 (0.61-1.33) | 1.01 (0.72-1.42) | 1.11 (1.00-1.25) |
| 20-25 | 0.72 (0.67-0.78) | 1.30 (1.20-1.41) | 0.72 (0.53-0.98) | 1.39 (1.01-1.92) | 0.82 (0.62-1.08) | 1.30 (1.20-1.41) |
|  |  |  |  |  |  |  |
| **Employment** | N=60,047 | N=59,541 | N=2,593 | N=2,461 | N=2,520 | N=2,449 |
| **Unemployed** |  |  |  |  |  |  |
| **Sex** | | | | | | |
| Men | 2.00 (1.82-2.19) | 1.72 (1.56-1.89) | 2.16 (1.61-2.92) | 2.17 (1.58-2.96) | 1.35 (1.00-1.84) | 1.23 (0.89-1.68) |
| Women | 1.74 (1.58-1.92) | 1.48 (1.35-1.63) | 1.21 (0.90-1.62) | 1.12 (0.82-1.54) | 1.39 (1.03-1.87) | 1.24 (0.91-1.70) |
| **Age of probands at baseline, years** | | | | | | |
| 5-14 | 1.56 (1.02-2.37) | 1.40 (0.90-2.17) | 1.20 (0.40-3.60) | 1.82 (0.29-11.19) | 2.84 (0.84-9.61) | 2.04 (0.39-10.67) |
| 15-19 | 1.74 (1.55-1.96) | 1.53 (1.35-1.72) | 1.60 (1.13-2.26) | 1.59 (1.10-2.31) | 1.16 (0.82-1.64) | 1.22 (0.84-1.76) |
| 20-25 | 1.95 (1.79-2.13) | 1.65 (1.51-1.80) | 1.68 (1.29-2.19) | 1.54 (1.16-2.05) | 1.43 (1.07-1.90) | 1.23 (0.91-1.66) |
|  |  |  |  |  |  |  |
| **Income (median, EUR) ^*^** | N=60,097 | N=59,541 | N=2,545 | N=2,461 | N=2,563 | N=2,449 |
| **Disposable income (top-coded)** | | | | | | |
| **Sex** | | | | | | |
| Men | -3281.9 (-3881.3,  -2682.6) | -2664.0 (-3179.5,  -2148.5) | -2752.6 (-4592.4, -912.8) | -2460.9 (-4016.2,  -905.6) | -1337.4 (-3361.4, 686.6) | -2309.2 (-3908.3,  -710.1) |
| Women | -1646.4 (-2047.3,  -1245.4) | -1275.2 (-1670.2,  -880.2) | -837.5 (-2044.2, 369.2) | -1041.2 (-2358.3, 275.9) | -340.2 (-1537.3, 686.6) | -697.4 (-1918.7, 524.0) |
| **Age of probands at baseline, years** | | | | | | |
| 5-14 | -1159.3 (-2888.0, 569.3) | -2791.3 (-4016.9,  -1565.7) | 1429.4 (-5092.9, 7951.7) | -1008.7 (-5506.5, 3489.0) | -5419.4 (-12500.5, 1661.8) | -5373.6 (-16450, 5702.4) |
| 15-19 | -2360.3 (-2957.9,  -1762.6) | -1733.6 (-2314.6,  -1152.6) | -2189.6 (-4007.8, -371.4) | -1529.8 (-3263.6, 204.0) | 418.9 (-1318.9, 2156.6) | -1350.4 (-3148.0, 447.4) |
| 20-25 | -2500.7 (-2923.9,  -2077.6) | -2095.6 (-2530.8,  -1660.4) | -1373.2 (-2621.8, -124.6) | -1988.5 (-3041.6,  -935.4) | -1094.1 (-2585.0, 396.9) | -1401.3 (-2803.8, 1.2) |

The reference group was the siblings of unaffected probands.

Adjusted: sex, age, residential region at baseline, any MPD diagnosis in the siblings before baseline, paternal and maternal highest education level at baseline, paternal and maternal history of any mental disorders before baseline, paternal and maternal age at birth of siblings of the affected and unaffected probands and the square of paternal and maternal age. Sex was not adjusted in the subgroup analysis by siblings’ sex.

The estimates for education achievement and employment are ORs and 95% CI.

**^*^** The estimates for income are coefficient and 95%CI.

# Table S6. Associations between having an affected proband with *bipolar disorder* and subsequent SES at the end of follow-up across different subgroups (End-point analysis).

|  | **Full siblings** | | **Maternal half-siblings** | | **Paternal half-siblings** | |
| --- | --- | --- | --- | --- | --- | --- |
|  | Crude | Adjusted | Crude | Adjusted | Crude | Adjusted |
| **Education** | N=19,802 | N=19,656 | N=808 | N=748 | N=990 | N=953 |
| **Not having a higher education degree** | | | | | | |
| **Sex** | | | | | | |
| Men | 0.74 (0.64-0.87) | 1.23 (1.04-1.46) | 0.69 (0.36-1.29) | 1.82 (0.89-3.75) | 1.12 (0.68-1.86) | 1.23 (1.04-1.46) |
| Women | 0.76 (0.66-0.88) | 1.29 (1.11-1.51) | 0.98 (0.61-1.57) | 0.90 (0.52-1.59) | 1.00 (0.64-1.55) | 1.29 (1.11-1.51) |
| **Age of probands at baseline, years** | | | | | | |
| 5-14 | 0.48 (0.18-1.25) | 2.62 (0.66-10.35) | NA | NA | NA | NA |
| 15-19 | 0.76 (0.59-0.98) | 1.32 (1.01-1.72) | 1.39 (0.61-3.15) | 1.03 (0.39-2.72) | 2.15 (1.09-4.26) | 1.32 (1.01-1.72) |
| 20-25 | 0.77 (0.69-0.87) | 1.25 (1.09-1.43) | 0.83 (0.54-1.26) | 1.29 (0.81-2.06) | 0.86 (0.60-1.23) | 1.25 (1.09-1.43) |
|  |  |  |  |  |  |  |
| **Employment** | N=18,148 | N=18,018 | N=729 | N=681 | N=900 | N=872 |
| **Unemployed** | | | | | | |
| **Sex** | | | | | | |
| Men | 1.83 (1.55-2.15) | 1.55 (1.31-1.84) | 2.27 (1.35-3.83) | 2.90 (1.60-5.26) | 1.44 (0.90-2.31) | 1.22 (0.73-2.05) |
| Women | 1.74 (1.47-2.05) | 1.49 (1.26-1.77) | 1.25 (0.75-2.06) | 1.27 (0.70-2.32) | 1.83 (1.11-3.01) | 2.08 (1.20-3.61) |
| **Age of probands at baseline, years** | | | | | | |
| 5-14 | 2.87 (1.18-6.97) | 4.19 (0.75-23.41) | NA | NA | NA | NA |
| 15-19 | 1.74 (1.33-2.29) | 1.62 (1.23-2.14) | 1.78 (0.65-4.90) | 1.30 (0.26-6.37) | 0.97 (0.45-2.10) | 0.71 (0.26-1.95) |
| 20-25 | 1.78 (1.56-2.03) | 1.50 (1.31-1.71) | 1.60 (1.10-2.33) | 1.95 (1.29-2.94) | 1.79 (1.20-2.67) | 1.63 (1.08-2.48) |
|  |  |  |  |  |  |  |
| **Income (median, EUR) ^*^** | N=18,226 | N=18,018 | N=738 | N=681 | N=887 | N=872 |
| **Disposable income (top-coded)** | | | | | | |
| **Sex** | | | | | | |
| Men | -2506.6 (-3624.7,  -1388.5) | -1550.7 (-2600.2,  -501.1) | -3635.2 (-9256.0, 1985.7) | -5404.1 (-9200.5,  -1607.7) | -2422.5 (-5277.2, 432.2) | -1258.5 (-4648.7, 2131.8) |
| Women | -520.1 (-1244.7, 204.4) | -1145.4 (-1994.0,  -296.7) | 2396.7 (235.6, 4557.8) | 119.0 (-2051.4, 2289.4) | -680.1 (-3841.5, 2481.3) | -367.5 (-2563.2, 1828.2) |
| **Age of probands at baseline, years** | | | | | | |
| 5-14 | -3548.6 (-9475.5, 2378.4) | -3626.9 (-10186,  -2932.3) | NA | NA | NA | NA |
| 15-19 | -1946.2 (-3025.9, -866.5) | -3113.8 (-4658.4,  -1569.3) | 1532.8 (-3201.0, 6266.6) | -2982.7 (-8161.8, 2196.4) | -1512.0 (-5921.0, 2896.9) | 5155.8 (418.4, 9893.3) |
| 20-25 | -1612.4 (-2275.0, -949.7) | -1189.5 (-1951.3, -427.7) | -75.5 (-2495.2, 2344.1) | -655.1 (-2304.6, 994.4) | -1250.2 (-3641.5, 1141.1) | -2097.3 (-4066.6, -128.0) |

The reference group was the siblings of unaffected probands.

Adjusted: sex, age, residential region at baseline, any MPD diagnosis in the siblings before baseline, paternal and maternal highest education level at baseline, paternal and maternal history of any mental disorders before baseline, paternal and maternal age at birth of siblings of the affected and unaffected probands and the square of paternal and maternal age. Sex was not adjusted in the subgroup analysis by siblings’ sex.

The estimates for education achievement and employment are ORs and 95% CI.

**^*^** The estimates for income are coefficient and 95%CI.

# Table S7. Associations between having an affected proband with *depressive disorders* and subsequent SES at the end of follow-up across different subgroups (End-point analysis).

|  | **Full siblings** | | **Maternal half-siblings** | | **Paternal half-siblings** | |
| --- | --- | --- | --- | --- | --- | --- |
|  | Crude | Adjusted | Crude | Adjusted | Crude | Adjusted |
| ***Depressive disorders*** |  |  |  |  |  |  |
| **Education** | N=208,709 | N=207,224 | N=8,783 | N=8,218 | N=8,645 | N=8,353 |
| **Not having a higher education degree** | | | | | | |
| **Sex** | | | | | | |
| Men | 0.68 (0.65-0.71) | 1.28 (1.22-1.34) | 0.89 (0.74-1.07) | 0.97 (0.80-1.19) | 0.71 (0.59-0.85) | 1.28 (1.22-1.34) |
| Women | 0.69 (0.66-0.72) | 1.26 (1.20-1.32) | 0.77 (0.65-0.90) | 1.10 (0.92-1.31) | 0.87 (0.75-1.00) | 1.26 (1.20-1.32) |
| **Age of probands at baseline, years** | | | | | | |
| 5-14 | 0.50 (0.42-0.59) | 1.66 (1.38-1.98) | 0.67 (0.43-1.04) | 1.50 (0.88-2.56) | 0.68 (0.45-1.02) | 1.66 (1.38-1.98) |
| 15-19 | 0.71 (0.67-0.75) | 1.25 (1.18-1.33) | 0.89 (0.73-1.07) | 0.89 (0.72-1.11) | 0.91 (0.75-1.10) | 1.25 (1.18-1.33) |
| 20-25 | 0.70 (0.67-0.74) | 1.25 (1.19-1.31) | 0.80 (0.68-0.95) | 1.10 (0.92-1.33) | 0.77 (0.66-0.90) | 1.25 (1.19-1.31) |
|  |  |  |  |  |  |  |
| **Employment** | N=191,650 | N=190,281 | N=8,185 | N=7,663 | N=8,122 | N=7,859 |
| **Unemployed** | | | | | | |
| **Sex** | | | | | | |
| Men | 1.73 (1.64-1.82) | 1.45 (1.37-1.53) | 1.53 (1.31-1.79) | 1.35 (1.15-1.60) | 1.17 (0.99-1.38) | 1.04 (0.87-1.24) |
| Women | 1.48 (1.41-1.57) | 1.27 (1.20-1.34) | 1.36 (1.15-1.60) | 1.19 (1.00-1.42) | 1.38 (1.17-1.63) | 1.19 (1.00-1.42) |
| **Age of probands at baseline, years** | | | | | | |
| 5-14 | 1.96 (1.63-2.35) | 1.71 (1.41-2.07) | 1.35 (0.88-2.06) | 1.27 (0.78-2.08) | 1.73 (1.14-2.61) | 1.74 (1.09-2.79) |
| 15-19 | 1.51 (1.41-1.61) | 1.32 (1.23-1.40) | 1.36 (1.13-1.63) | 1.17 (0.96-1.42) | 1.16 (0.94-1.43) | 1.04 (0.83-1.29) |
| 20-25 | 1.64 (1.56-1.72) | 1.35 (1.28-1.43) | 1.52 (1.31-1.77) | 1.35 (1.16-1.59) | 1.28 (1.10-1.49) | 1.11 (0.94-1.30) |
|  |  |  |  |  |  |  |
| **Income (median, EUR) ^*^** | N=191,477 | N=190,284 | N=8,170 | N=7,663 | N=8,043 | N=7,859 |
| **Disposable income (top-coded)** | | | | | | |
| **Sex** | | | | | | |
| Men | -2607.3 (-2906.3,  -2308.3) | -1585.5 (-1856.4,  -1314.6) | -1808.1 (-2718.4,  -897.7) | -1088.7 (-1937.1,  -240.2) | -1887.4 (-2800.4,  -974.3) | -811.6 (-1680.1, 56.9) |
| Women | -1290.6 (-1524.4,  -1056.9) | -810.3 (-1056.4,  -564.2) | -1155.1 (-1797.6,  -512.6) | -613.5 (-1292.1, 65.1) | -887.6 (-1600.7,  -174.5) | -1339.6 (-2070.0,  -609.2) |
| **Age of probands at baseline, years** | | | | | | |
| 5-14 | -2915.3 (-3647.4,  -2183.2) | -2119.5 (-2921.7,  -1317.3) | -2241.7 (-4111.7,  -371.7) | -1703.8 (-3936.3, 528.7) | -2812.4 (-5139.0,  -485.9) | -2290.7 (-4688.3, 106.9) |
| 15-19 | -1739.2 (-2019.6,  -1458.7) | -1094.8 (-1386.4,  -803.2) | -1628.3 (-2518.0,  -738.5) | -774.1 (-1643.2, 95.1) | -1279.2 (-2254.7,  -303.6) | -1026.1 (-1900.9,  -151.4) |
| 20-25 | -2016.8 (-2242.7,  -1791.0) | -1193.6 (-1428.8,  -958.5) | -1259.7 (-1963.2,  -556.3) | -587.9 (-1340.1, 164.4) | -1268.5 (-1988.1,  -549.0) | -887.5 (-1658.0,  -117.1) |

The reference group was the siblings of unaffected probands.

Adjusted: sex, age, residential region at baseline, any MPD diagnosis in the siblings before baseline, paternal and maternal highest education level at baseline, paternal and maternal history of any mental disorders before baseline, paternal and maternal age at birth of siblings of the affected and unaffected probands and the square of paternal and maternal age. Sex was not adjusted in the subgroup analysis by siblings’ sex.

The estimates for education achievement and employment are ORs and 95% CI.

**^*^**The estimates for income are coefficient and 95%CI.

# Table S8. Associations between having an affected proband with *anxiety disorders* and subsequent SES at the end of follow-up across different subgroups (End point analysis).

|  | **Full siblings** | | **Maternal half-siblings** | | **Paternal half-siblings** | |
| --- | --- | --- | --- | --- | --- | --- |
|  | Crude | Adjusted | Crude | Adjusted | Crude | Adjusted |
| ***Anxiety disorders*** |  |  |  |  |  |  |
| **Education** | N=255,997 | N=253,968 | N=11,261 | N=10,607 | N=10,994 | N=10,676 |
| **Not having a higher education degree** | | | | | | |
| **Sex** | | | | | | |
| Men | 0.67 (0.64-0.70) | 1.25 (1.20-1.31) | 0.71 (0.60-0.84) | 1.21 (1.02-1.45) | 0.70 (0.59-0.82) | 1.25 (1.20-1.31) |
| Women | 0.63 (0.60-0.66) | 1.35 (1.29-1.41) | 0.64 (0.56-0.74) | 1.34 (1.15-1.56) | 0.77 (0.68-0.89) | 1.35 (1.29-1.41) |
| **Age of probands at baseline, years** | | | | | | |
| 5-14 | 0.49 (0.43-0.56) | 1.67 (1.46-1.90) | 0.47 (0.35-0.64) | 1.81 (1.29-2.54) | 0.55 (0.41-0.73) | 1.67 (1.46-1.90) |
| 15-19 | 0.65 (0.62-0.68) | 1.31 (1.25-1.38) | 0.65 (0.55-0.78) | 1.26 (1.05-1.52) | 0.75 (0.64-0.88) | 1.31 (1.25-1.38) |
| 20-25 | 0.70 (0.67-0.73) | 1.25 (1.19-1.31) | 0.77 (0.66-0.90) | 1.19 (1.00-1.40) | 0.81 (0.69-0.95) | 1.25 (1.19-1.31) |
|  |  |  |  |  |  |  |
| **Employment** | N=235,072 | N=233,293 | N=10,350 | N=9,727 | N=10,113 | N=9,815 |
| **Unemployed** | | | | | | |
| **Sex** | | | | | | |
| Men | 1.66 (1.58-1.74) | 1.40 (1.33-1.47) | 1.49 (1.29-1.72) | 1.29 (1.11-1.50) | 1.30 (1.12-1.51) | 1.18 (1.01-1.37) |
| Women | 1.53 (1.46-1.61) | 1.34 (1.28-1.41) | 1.36 (1.18-1.57) | 1.19 (1.03-1.39) | 1.22 (1.05-1.42) | 1.12 (0.96-1.31) |
| **Age of probands at baseline, years** | | | | | | |
| 5-14 | 1.98 (1.75-2.24) | 1.73 (1.52-1.96) | 1.40 (1.05-1.85) | 1.20 (0.89-1.61) | 1.41 (1.04-1.90) | 1.29 (0.93-1.77) |
| 15-19 | 1.54 (1.46-1.63) | 1.35 (1.28-1.43) | 1.36 (1.16-1.60) | 1.20 (1.02-1.42) | 1.39 (1.17-1.65) | 1.26 (1.06-1.51) |
| 20-25 | 1.59 (1.51-1.67) | 1.33 (1.26-1.40) | 1.49 (1.28-1.72) | 1.29 (1.11-1.51) | 1.13 (0.97-1.32) | 1.02 (0.86-1.20) |
|  |  |  |  |  |  |  |
| **Income (median, EUR) ^*^** | N=235,019 | N=233,307 | N=10,443 | N=9,727 | N=10,162 | N=9,815 |
| **Disposable income (top-coded)** | | | | | | |
| **Sex** |  |  |  |  |  |  |
| Men | -2702.1 (-2957.3,  -2446.8) | -1506.7 (-1772.6,  -1240.8) | -1857.9 (-2615.2,  -1100.6) | -1018.9 (-1858.8,  -179.0) | -1858.2 (-2715.9,  -1000.5) | -1737.8 (-2732.3,  -743.3) |
| Women | -1370.1 (-1558.1,  -1182.0) | -929.8 (-1139.3,  -720.3) | -485.8 (-1088.2, 116.6) | -573.2 (-1142.7,  -3.7) | -784.2 (-1317.9,  -250.6) | -474.6 (-1067.3, 103.7) |
| **Age of probands at baseline, years** | | | | | | |
| 5-14 | -2772.9 (-3358.3,  -2187.6) | -2260.3 (-2888.1,  -1632.4) | -828.8 (-2032.2, 374.7) | -377.5 (-1731.0, 976.0) | -2712.2 (-4145.4,  -1279.0) | -1031.9 (-2257.5, 193.6) |
| 15-19 | -2064.0 (-2336.8,  -1791.2) | -1004.0 (-1273.1,  -735.0) | -1111.6 (-1962.9,  -260.3) | -880.4 (-1587.8,  -173.0) | -1232.8 (-2075.1,  -390.5) | -1090.5 (-1941.1,  -240.0) |
| 20-25 | -1957.0 (-2173.6,  -1740.4) | -1133.5 (-1366.3,  -900.7) | -1230.3 (-1908.7,  -551.9) | -646.6 (-1338.7, 45.4) | -970.7 (-1613.2,  -328.1) | -734.2 (-1510.8, 42.4) |

The reference group was the siblings of unaffected probands.

Adjusted: sex, age, residential region at baseline, any MPD diagnosis in the siblings before baseline, paternal and maternal highest education level at baseline, paternal and maternal history of any mental disorders before baseline, paternal and maternal age at birth of siblings of the affected and unaffected probands and the square of paternal and maternal age. Sex was not adjusted in the subgroup analysis by siblings’ sex.

The estimates for education achievement and employment are ORs and 95% CI.

**^*^** The estimates for income are coefficient and 95%CI.

# Table S9. Associations between having an affected proband with schizophrenia spectrum disorders and subsequent SES over the observation period across different subgroups (Longitudinal analysis).

|  | **Full siblings** | | **Maternal half-siblings** | | **Paternal half-siblings** | |
| --- | --- | --- | --- | --- | --- | --- |
|  | Crude | Adjusted | Crude | Adjusted | Crude | Adjusted |
| ***Schizophrenia spectrum disorders*** |  |  |  |  |  |  |
| **Employment** | N=100,970 | N=100,084 | N=4,081 | N=3,869 | N=4,112 | N=3,982 |
| **Unemployed** | | | | | | |
| **Sex** | | | | | | |
| Men | 1.64 (1.57-1.70) | 1.51 (1.44-1.57) | 1.47 (1.27-1.71) | 1.46 (1.25-1.70) | 1.35 (1.17-1.56) | 1.28 (1.11-1.48) |
| Women | 1.44 (1.38-1.50) | 1.36 (1.31-1.42) | 1.25 (1.10-1.43) | 1.19 (1.05-1.36) | 1.19 (1.05-1.35) | 1.17 (1.03-1.33) |
| **Age of probands at baseline, years** | | | | | | |
| 5-14 | 1.39 (1.22-1.57) | 1.34 (1.19-1.51) | 1.15 (0.75-1.75) | 0.90 (0.59-1.37) | 1.97 (1.24-3.11) | 1.30 (0.82-2.08) |
| 15-19 | 1.40 (1.34-1.47) | 1.36 (1.30-1.42) | 1.32 (1.12-1.56) | 1.36 (1.15-1.61) | 1.08 (0.92-1.26) | 1.13 (0.96-1.31) |
| 20-25 | 1.64 (1.58-1.70) | 1.49 (1.43-1.54) | 1.39 (1.22-1.59) | 1.31 (1.15-1.49) | 1.37 (1.20-1.56) | 1.27 (1.11-1.44) |
|  |  |  |  |  |  |  |
| **Income^*^** | N=100,964 | N=100,073 | N=4,081 | N=3,869 | N=4,112 | N=3,982 |
| **Disposable income (top-coded)** | | | | | | |
| **Sex** | | | | | | |
| Men | -2745.5 (-3079.9,  -2411.5) | -2046.4 (  -2335.5,  -1757.4) | -1521.7 (-2576.0,  -467.4) | -1371.9 (-2213.7, -530.1) | -1329.5 (-2483.8,  -175.3) | -878.9 (-1822.3, 64.4) |
| Women | -1251.9 (-1504.0,  -999.8) | -969.1 (-1182.3,  -755.8) | -962.5 (-1767.0,  -158.0) | -545.3 (-1164.8, 74.2) | -205.3 (-1055.8, 645.2) | 124.8 (-521.7, 771.2) |
| **Age of probands at baseline, years** | | | | | | |
| 5-14 | -2157.2 (-3178.1,  -1136.4) | -1492.5 (-2353.4,  -631.6) | -352.3 (-3162.5, 2457.8) | 1637.0 (-390.8, 3664.9) | -3106.9 (-6709.1, 495.4) | 104.8 (-2992.2, 3201.9) |
| 15-19 | -1175.5 (-1519.1,  -831.9) | -1043.6 (-1335.4,  -751.8) | -1135.9 (-2226.2, -45.7) | -905.0 (-1794.2,  -15.7) | 82.5 (-979.7, 1144.8) | -162.6 (-986.4, 661.3) |
| 20-25 | -2482.5 (-2754.3,  -2210.8) | -1824.7 (-2061.1,  -1588.2) | -1349.7 (-2211.2, -488.1) | -1212.8 (-1885.4, -540.2) | -1256.3 (-2227.7,  -284.8) | -329.9 (-1105.1, 445.3) |
| ***Earnings^**^*** | | | | | | |
| **Sex** | | | | | | |
| Men | -5858.9 (-6514.8,  -5203.1) | -4374.7 (-4947.2,  -3802.2) | -3908.0 (-5963.3, -1852.7) | -2774.3 (-4701.1, -847.4) | -3462.2 (-5675.4,  -1249.0) | -2111.1 (-4087.9, -134.4) |
| Women | -2733.1 (-2891.4,  -2574.9) | -1957.6 (-2112.3,  -1802.9) | -2652.1 (-3932.5, -1371.8) | -1834.5 (-2966.3, -702.8) | -1382.3 (-2694.7,  -70.0) | -895.7 (-2013.1, 221.8) |
| **Age of probands at baseline, years** | | | | | | |
| 5-14 | -3216.9 (-5009.4,  -1424.3) | -2361.5 (-3985.3,  -737.8) | 28.6 (-4632.9, 4690.2) | 3617.7 (-185.2, 7420.6) | -4819.2 (-12314.7, 2676.3) | 357.1 (-5743.2, 6457.5) |
| 15-19 | -3146.4 (-3778.3,  -2514.4) | -2639.0 (-3187.2,  -2090.7) | -3157.5 (-5140.4, -1174.6) | -1690.4 (-3819.3, -438.5) | -598.5 (-2610.8, 1413.8) | -442.2 (-2222.1, 1337.7) |
| 20-25 | -4776.7 (-4961.2,  -4592.1) | -3456.1 (-3641.5,  -3270.8) | -3675.0 (-5295.4, -2054.6) | -3089.5 (-4437.4, -1741.5) | -3775.9 (-5464.2, -2087.6) | -1902.1 (-3348.2, -456.0) |

The reference group was the siblings of unaffected probands.

Adjusted: sex, age, residential region at baseline, any MPD diagnosis in the siblings before baseline, paternal and maternal highest education level at baseline, paternal and maternal history of any mental disorders before baseline, paternal and maternal age at birth of siblings of the affected and unaffected probands and the square of paternal and maternal age. Sex was not adjusted in the subgroup analysis by siblings’ sex.

The estimates for employment are ORs and 95% CI.

**^*^**The estimates for income are mean and 95%CI.

^**^Additional analysis

# Table S10. Associations between having an affected proband with bipolar disorders and subsequent SES over the observation period across different subgroups (Longitudinal analysis).

|  | **Full siblings** | | **Maternal half-siblings** | | **Paternal half-siblings** | |
| --- | --- | --- | --- | --- | --- | --- |
|  | Crude | Adjusted | Crude | Adjusted | Crude | Adjusted |
| ***Bipolar disorders*** |  |  |  |  |  |  |
| **Employment** | N=30,471 | N=30,228 | N=1,185 | N=1,100 | N=1,451 | N=1,401 |
| **Unemployed** | | | | | | |
| **Sex** | | | | | | |
| Men | 1.64 (1.51-1.78) | 1.42 (1.30-1.54) | 1.55 (1.15-2.08) | 1.52 (1.12-2.05) | 1.34 (1.05-1.71) | 1.03 (0.81-1.31) |
| Women | 1.36 (1.26-1.46) | 1.21 (1.13-1.31) | 1.18 (0.91-1.54) | 1.25 (0.96-1.62) | 1.30 (1.02-1.65) | 1.22 (0.96-1.56) |
| **Age of probands at baseline, years** | | | | | | |
| 5-14 | 1.55 (1.10-2.19) | 1.85 (1.28-2.66) | 1.56 (0.19-12.6) | NA | NA | NA |
| 15-19 | 1.42 (1.27-1.60) | 1.35 (1.20-1.52) | 1.46 (0.97-2.21) | 1.52 (0.95-2.43) | 1.31 (0.92-1.84) | 1.00 (0.70-1.42) |
| 20-25 | 1.50 (1.41-1.60) | 1.30 (1.21-1.38) | 1.32 (1.05-1.64) | 1.31 (1.05-1.63) | 1.31 (1.08-1.60) | 1.14 (0.94-1.39) |
|  |  |  |  |  |  |  |
| **Income^*^** | N=30,470 | N=30,227 | N=1,185 | N=1,100 | N=1,451 | N=1,401 |
| **Disposable income (top-coded)** | | | | | | |
| **Sex** | | | | | | |
| Men | -2734.7 (-3382.8, -2086.6) | -1733.9 (-2285.7, -1182.1) | -1500.4 (-3711.3, 710.5) | -1027.0 (-2752.8, 698.7) | -2612.9 (-4531.5, -694.3) | -858.0 (-2364.4, 648.4) |
| Women | -897.5 (-1354.0, -441.0) | -683.5 (-1073.1, -293.9) | 342.5 (-1154.7, 1839.8) | 941.0 (-275.9, 2157.9) | -18.3 (-1533.8, 1497.1) | 15.8 (-1185.1, 1216.6) |
| **Age of probands at baseline, years** | | | | | | |
| 5-14 | -2085.5 (-5487.3, 1316.4) | -3765.8 (-7019.4, -512.2) | -2443.4 (-15659.6, 10772.8) | 27048.4 (4494.1, 49602.8) | -855.3 (-1897.6, 187.1) | -981.8 (-2053.7, 90.0) |
| 15-19 | -1355.7 (-2194.4, -516.9) | -1350.8 (-2076.9, -624.7) | -2339.4 (-5100.6, 421.7) | -2250.6 (-4354.0, -147.3) | -2504.1 (-5145.2, 137.1) | -50.8 (-2158.9, 2057.3) |
| 20-25 | -1902. 0 (-2346.2, -1457.7) | -1196.8 (-1580.4, -813.3) | -199.3 (-1649.8, 1251.3) | 327.9 (-861.7, 1517.6) | -1029.1 (-2399.2, 341.0) | -411.1 (-1492.6, 670.4) |
| ***Earnings^**^*** | | | | | | |
| **Sex** | | | | | | |
| Men | -5098.6 (-6668.5, -3528.7) | -3164.0 (-4505.7, -1822.3) | -3150.4 (-7634.2, 1333.4) | -2227.7 (-5847.4, 1392.0) | -5676.4 (-9134.4, -2218.5) | -2254.6 (-5041.4, -532.2) |
| Women | -2419.5 (-3232.9, -1606.1) | -1635.1 (-2351.3, -918.8) | -836.2 (-3331.7, 1659.3) | -537.6 (-2742.6, 1667.3) | -1608.3 (-4178.3, 961.6) | -1138.4 (-3325.0, 1048.2) |
| **Age of probands at baseline, years** | | | | | | |
| 5-14 | -3781.1 (-10005.4, 2443.3) | -5772.8 (-11385.4, -160.2) | -5310.4 (-27575, 16954.1) | NA | -2303.1 (-4254.3, -352.0) | -2283.0 (-4240.0, -325.9) |
| 15-19 | -3277.7 (-4785.8, -1769.5) | -2873.9 (-4215.0, -1532.8) | -4785.1 (-9454.0, -116.2) | -4204.4 (-8261.8, -147.0) | -4696.3 (-9310.9, -81.7) | -820.3 (-4869.0, 3228.4) |
| 20-25 | -3977.3 (-5026.6, -2928.0) | -2328.0 (-3229.4, -1426.6) | -1675.0 (-4508.5, 1158.4) | -889.1 (-3284.6, 1506.5) | -3401.3 (-5839.7, -963.0) | -1767.1 (-3760.2, 226.1) |

The reference group was the siblings of unaffected probands.

Adjusted: sex, age, residential region at baseline, any MPD diagnosis in the siblings before baseline, paternal and maternal highest education level at baseline, paternal and maternal history of any mental disorders before baseline, paternal and maternal age at birth of siblings of the affected and unaffected probands and the square of paternal and maternal age. Sex was not adjusted in the subgroup analysis by siblings’ sex.

The estimates for employment are ORs and 95% CI.

**^*^**The estimates for income are mean and 95%CI.

^**^Additional analysis

# Table S11. Associations between having an affected proband with depressive disorders and subsequent SES over the observation period across different subgroups (Longitudinal analysis).

|  | **Full siblings** | | **Maternal half-siblings** | | **Paternal half-siblings** | |
| --- | --- | --- | --- | --- | --- | --- |
|  | Crude | Adjusted | Crude | Adjusted | Crude | Adjusted |
| ***Depressive disorders*** |  |  |  |  |  |  |
| **Employment** | N=320,877 | N=318,586 | N=13,051 | N=12,218 | N=12,908 | N=12,486 |
| **Unemployed** | | | | | | |
| **Sex** | | | | | | |
| Men | 1.41 (1.38-1.45) | 1.29 (1.26-1.33) | 1.36 (1.26-1.48) | 1.19 (1.10-1.29) | 1.20 (1.10-1.30) | 1.08 (0.99-1.17) |
| Women | 1.30 (1.27-1.33) | 1.22 (1.19-1.24) | 1.24 (1.15-1.34) | 1.10 (1.02-1.19) | 1.26 (1.17-1.36) | 1.18 (1.09-1.27) |
| **Age of probands at baseline, years** | | | | | | |
| 5-14 | 1.43 (1.33-1.53) | 1.35 (1.26-1.44) | 1.33 (1.12-1.58) | 1.10 (0.93-1.30) | 1.44 (1.20-1.73) | 1.36 (1.14-1.62) |
| 15-19 | 1.26 (1.23-1.30) | 1.21 (1.18-1.24) | 1.34 (1.23-1.46) | 1.13 (1.03-1.23) | 1.21 (1.11-1.33) | 1.09 (1.00-1.20) |
| 20-25 | 1.43 (1.40-1.46) | 1.28 (1.25-1.31) | 1.28 (1.18-1.38) | 1.16 (1.07-1.25) | 1.20 (1.12-1.30) | 1.11 (1.03-1.20) |
|  |  |  |  |  |  |  |
| **Income^*^** | N=320,873 | N=318,580 | N=13,047 | N=12,214 | N=12,905 | N=12,483 |
| **Disposable income (top-coded)** | | | | | | |
| **Sex** | | | | | | |
| Men | -2014.1 (-2197.7, -1830.5) | -1410.2 (-1564.9, -1255.5) | -1684.9 (-2249.5, -1120.3) | -690.6 (-1144.1, -237.1) | -1117.0 (-1746.1, -487.9) | -586.9 (-1079.9, -93.9) |
| Women | -799.4 (-935.9, -662.9) | -657.7 (-772.0, -543.4) | -666.5 (-1106.5, -226.4) | -187.5 (-526.4, 151.3) | -718.1 (-1193.0, -243.2) | -421.4 (-782.8, -60.0) |
| **Age of probands at baseline, years** | | | | | | |
| 5-14 | -1946.0 (-2430.3, -1461.8) | -1569.1 (-1977.8, -1160.4) | -1961.2 (-3112.8, -809.7) | -935.0 (-1898.1, 28.2) | -2335.4 (-3653.1, -1017.7) | -1368.0 (-2361.4, -374.7) |
| 15-19 | -1018.4 (-1197.5, -839.4) | -820.6 (-969.7, -671.5) | -1509.6 (-2096.8, -922.3) | -289.2 (-745.8, 167.5) | -919.3 (-1574.1, -264.5) | -255.8 (-761.3, 249.6) |
| 20-25 | -1706.7 (-1859.8, -1553.6) | -1123.8 (-1255.8, -991.7) | -858.6 (-1343.0, -374.2) | -397.7 (-785.4, -10.0) | -708.3 (-1233, -183.5) | -512.0 (-921.5, -102.5) |
| ***Earnings^**^*** | | | | | | |
| **Sex** | | | | | | |
| Men | -4306.9 (-4696.2, -3917.7) | -2961.5 (-3302.2, -2620.7) | -3425.3 (-4468.6, -2382.0) | -1707.7 (-2619.6, -795.9) | -2052.8 (-3238.8, -866.9) | -901.0 (-1885.3, -83.3) |
| Women | -2338.6 (-2571.2, -2106.1) | -1632.5 (-1840.6, -1424.3) | -1802.2 (-2507.4, -1097.0) | -813.3 (-1428.2, -198.4) | -2379.5 (-3200.4, -1558.5) | -1418.0 (-2094.1, -741.9) |
| **Age of probands at baseline, years** | | | | | | |
| 5-14 | -4193.6 (-5038.6, -3348.5) | -3388.2 (-4133.4, -2643.1) | -3590.6 (-5500.4, -1680.7) | -1702.5 (-3467.6, 62.5) | -3706.6 (-5836.6, -1576.7) | -2731.3 (-4589.2, -873.5) |
| 15-19 | -2643.2 (-2964.5, -2322.0) | -1977.2 (-2257.9, -1696.5) | -2509.8 (-3550.5, -1469.1) | -856.7 (-1757.3, -43.9) | -2250.1 (-3364.2, -1136.0) | -981.3 (-1912.0, -50.6) |
| 20-25 | -3888.2 (-4219.5, -3557.0) | -2463.3 (-2752.5, -2174.0) | -2651.1 (-3531.0, -1771.2) | -1489.5 (-2246.3, -732.6) | -2021.9 (-3058.5, -985.2) | -1128.4 (-1972.3, -284.6) |

The reference group was the siblings of unaffected probands.

Adjusted: sex, age, residential region at baseline, any MPD diagnosis in the siblings before baseline, paternal and maternal highest education level at baseline, paternal and maternal history of any mental disorders before baseline, paternal and maternal age at birth of siblings of the affected and unaffected probands and the square of paternal and maternal age. Sex was not adjusted in the subgroup analysis by siblings’ sex.

The estimates for employment are ORs and 95% CI.

**^*^**The estimates for income are mean and 95%CI.

^**^Additional analysis

# Table S12. Associations between having an affected proband with anxiety disorders and subsequent SES over the observation period across different subgroups (Longitudinal analysis).

|  | **Full siblings** | | **Maternal half-siblings** | | **Paternal half-siblings** | |
| --- | --- | --- | --- | --- | --- | --- |
|  | Crude | Adjusted | Crude | Adjusted | Crude | Adjusted |
| ***Anxiety disorders*** |  |  |  |  |  |  |
| **Employment** | N=393,141 | N=390,076 | N=16,683 | N=15,689 | N=16,387 | N=15,907 |
| **Unemployed** | | | | | | |
| **Sex** | | | | | | |
| Men | 1.35 (1.26-1.38) | 1.26 (1.23-1.28) | 1.30 (1.22-1.39) | 1.18 (1.10-1.27) | 1.14 (1.06-1.22) | 1.08 (1.01-1.16) |
| Women | 1.25 (1.27-1.28) | 1.19 (1.17-1.21) | 1.26 (1.19-1.35) | 1.12 (1.06-1.20) | 1.19 (1.11-1.27) | 1.16 (1.09-1.23) |
| **Age of probands at baseline, years** |  |  |  |  |  |  |
| 5-14 | 1.31 (1.26-1.38) | 1.30 (1.24-1.36) | 1.34 (1.20-1.50) | 1.13 (1.02-1.25) | 1.21 (1.08-1.36) | 1.22 (1.09-1.36) |
| 15-19 | 1.25 (1.22-1.27) | 1.19 (1.16-1.22) | 1.28 (1.19-1.37) | 1.19 (1.10-1.27) | 1.20 (1.11-1.29) | 1.12 (1.04-1.21) |
| 20-25 | 1.36 (1.33-1.39) | 1.24 (1.21-1.26) | 1.27 (1.18-1.36) | 1.14 (1.06-1.22) | 1.13 (1.05-1.21) | 1.08 (1.00-1.16) |
|  |  |  |  |  |  |  |
| **Income^*^** | N=393,086 | N=390,009 | N=16,673 | N=15,678 | N=16,380 | N=15,899 |
| **Disposable income (top-coded)** | | | | | | |
| **Sex** | | | | | | |
| Men | -1791.1 (-1957.3, -1625.0) | -1213.6 (-1353.7, -1073.5) | -1556.0 (-2043.0, -1068.9) | -787.9 (-1177.3, -398.6) | -1161.3 (-1700.6, -622.0) | -726.9 (-1147.7, -306.0) |
| Women | -706.0 (-827.7, -584.4) | -593.2 (-695.2, -491.1) | -863.0 (-1254.4, -471.5) | -320.8 (-611.4, -30.2) | -430.0 (-843.6, -16.4) | -327.4 (-640.4, -14.4) |
| **Age of probands at baseline, years** | | | | | | |
| 5-14 | -1708.1 (-2046.3, -1369.8) | -1558.3 (-1851.8, -1264.8) | -1369.3 (-2097.0, -641.5) | -319.7 (-885.2, 245.9) | -977.4 (-1795.5, -159.3) | -895.5 (-1560.0, -231.1) |
| 15-19 | -1046.9 (-1205.5, -888.2) | -747.7 (-880.4, -615.1) | -1072.8 (-1562.3, -583.3) | -583.1 (-962.5, -203.7) | -1155.8 (-1696.1, -615.5) | -590.5 (-998.3, -182.7) |
| 20-25 | -1447.7 (-1592.4, -1302.9) | -911.5 (-1035.7, -787.2) | -1255.6 (-1726.3, -785.0) | -608.2 (-981.2, -235.1) | -473.8 (-974.3, 26.7) | -244.5 (-637.8, 148.8) |
| ***Earnings^**^*** | | | | | | |
| **Sex** | | | | | | |
| Men | -3963.3 (-4276.1, -3650.5) | -2594.6 (-2870.0, -2319.2) | -3065.7 (-3949.4, -2182.1) | -1605.5 (-2368.9, -842.1) | -1998.4 (-3052.9, -944.0) | -984.1 (-1879.8, -88.5) |
| Women | -2126.3 (-2205.4, -2047.3) | -1448.3 (-1529.4, -1367.2) | -2158.2 (-2785.8, -1530.5) | -1128.3 (-1667.2, -589.4) | -1635.4 (-2283.8, -987.1) | -1030.6 (-1580.7, -480.4) |
| **Age of probands at baseline, years** | | | | | | |
| 5-14 | -3915.8 (-4567.3, -3262.9) | -3230.9 (-3796.4, -2665.3) | -2784.2 (-3985.8, -1582.5) | -1231.9 (-2328.6, -135.3) | -2093.9 (-3439.2, -748.6) | -1378.3 (-2578.4, -178.2) |
| 15-19 | -2554.8 (-2663.5, -2446.1) | -1558.1 (-1662.4, -1453.8) | -2522.8 (-3368.0, -1677.6) | -1453.7 (-2181.2, -726.1) | -2531.3 (-3442.0, -1620.7) | -1537.4 (-2293.7, -781.2) |
| 20-25 | -3344.4 (-3450.2, -3238.6) | -1996.3 (-2096.0, -1896.6) | -2666.2 (-3523.5, -1808.9) | -1413.6 (-2143.0, -684.2) | -1245.1 (-2248.8, -241.4) | -370.6 (1213.2, 472.1) |

The reference group was the siblings of unaffected probands.

Adjusted: sex, age, residential region at baseline, any MPD diagnosis in the siblings before baseline, paternal and maternal highest education level at baseline, paternal and maternal history of any mental disorders before baseline, paternal and maternal age at birth of siblings of the affected and unaffected probands and the square of paternal and maternal age. Sex was not adjusted in the subgroup analysis by siblings’ sex.

The estimates for employment are ORs and 95% CI.

**^*^**The estimates for income are mean and 95%CI.

^**^Additional analysis

# Figure S1. Study sample derivation, full sibling cohorts.

**
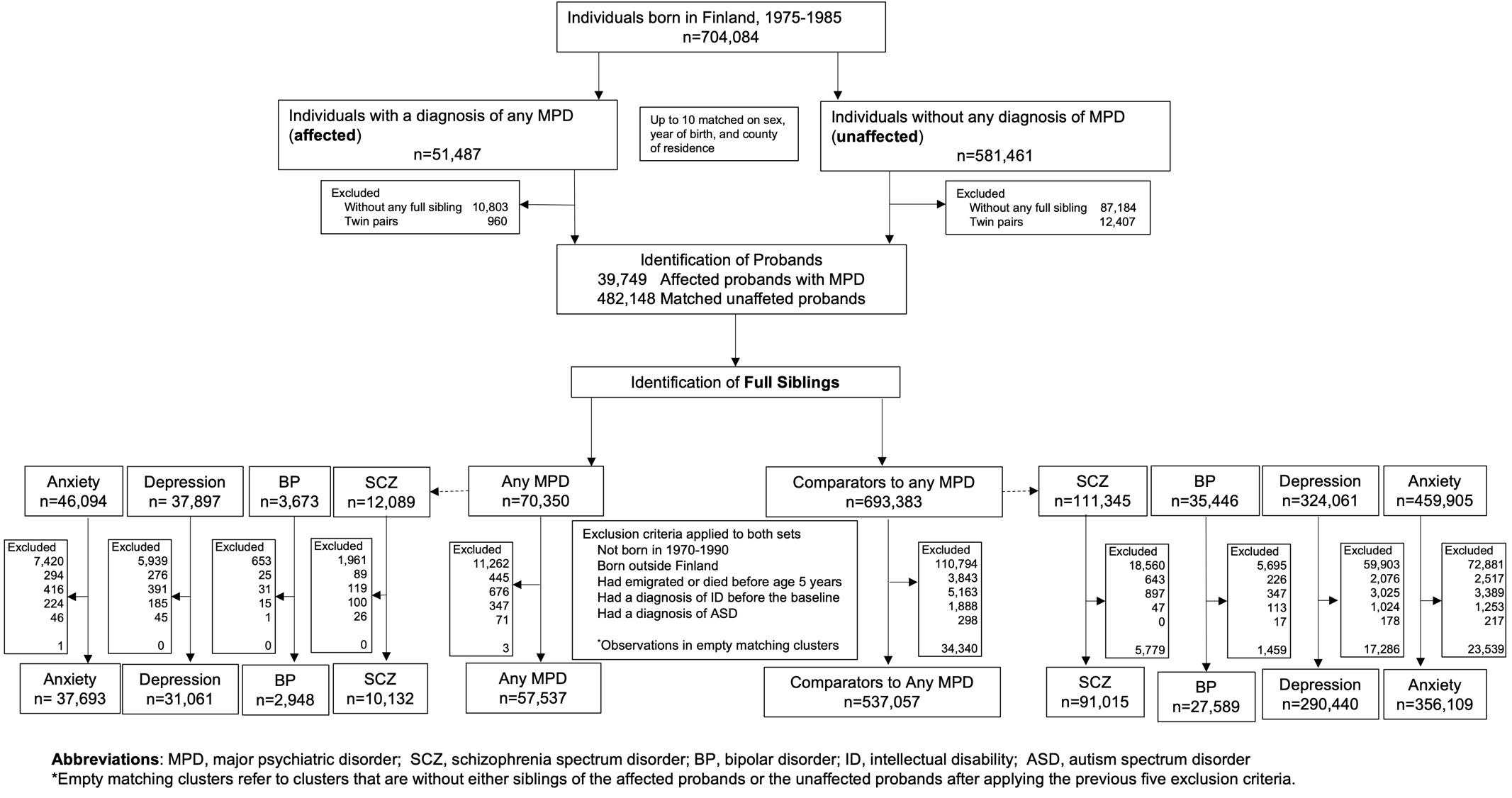
**

# Figure S2. Study sample derivation, maternal half sibling cohorts.

**
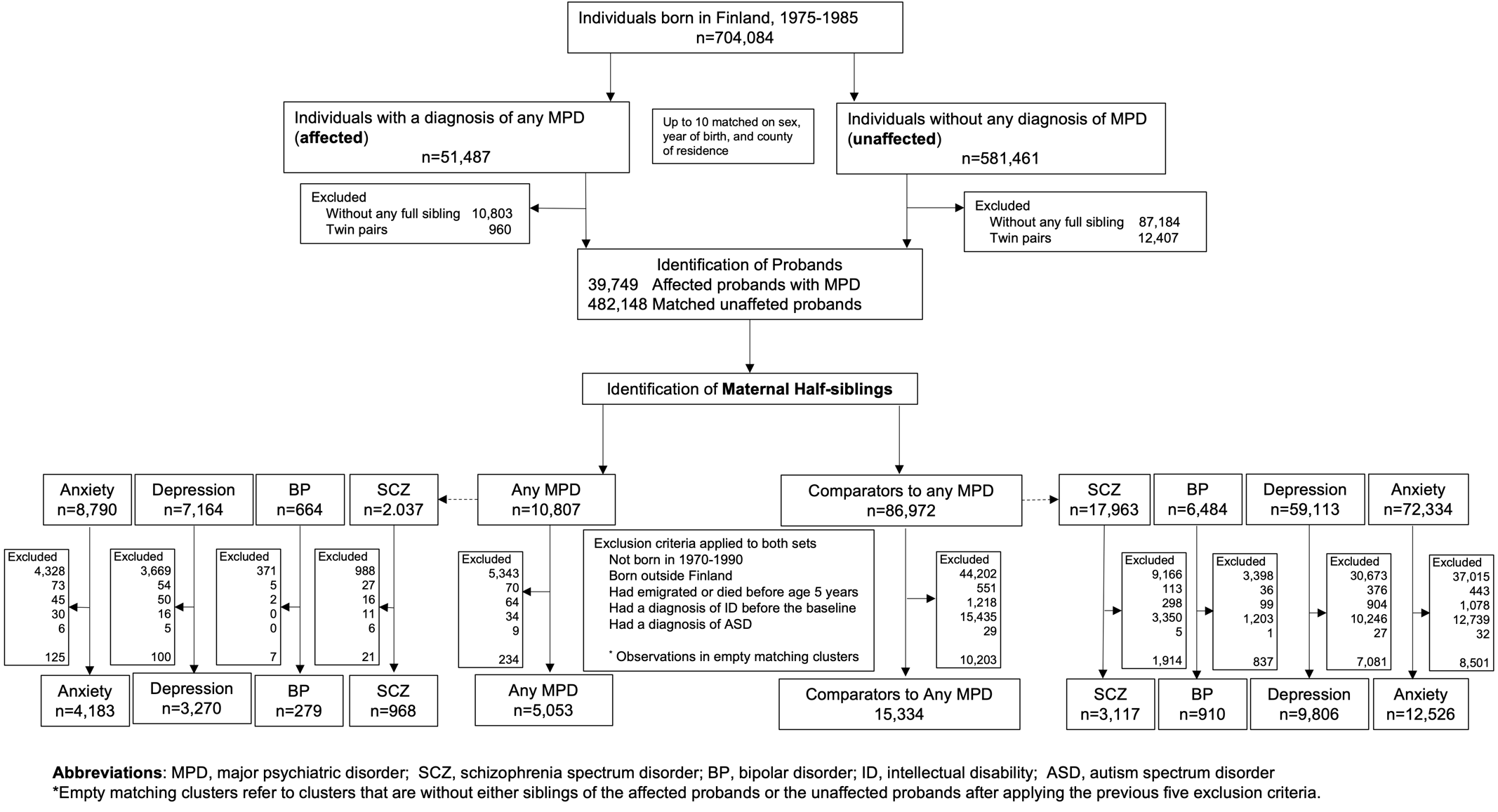
**

# Figure S3. Study sample derivation, paternal half sibling cohorts.

**
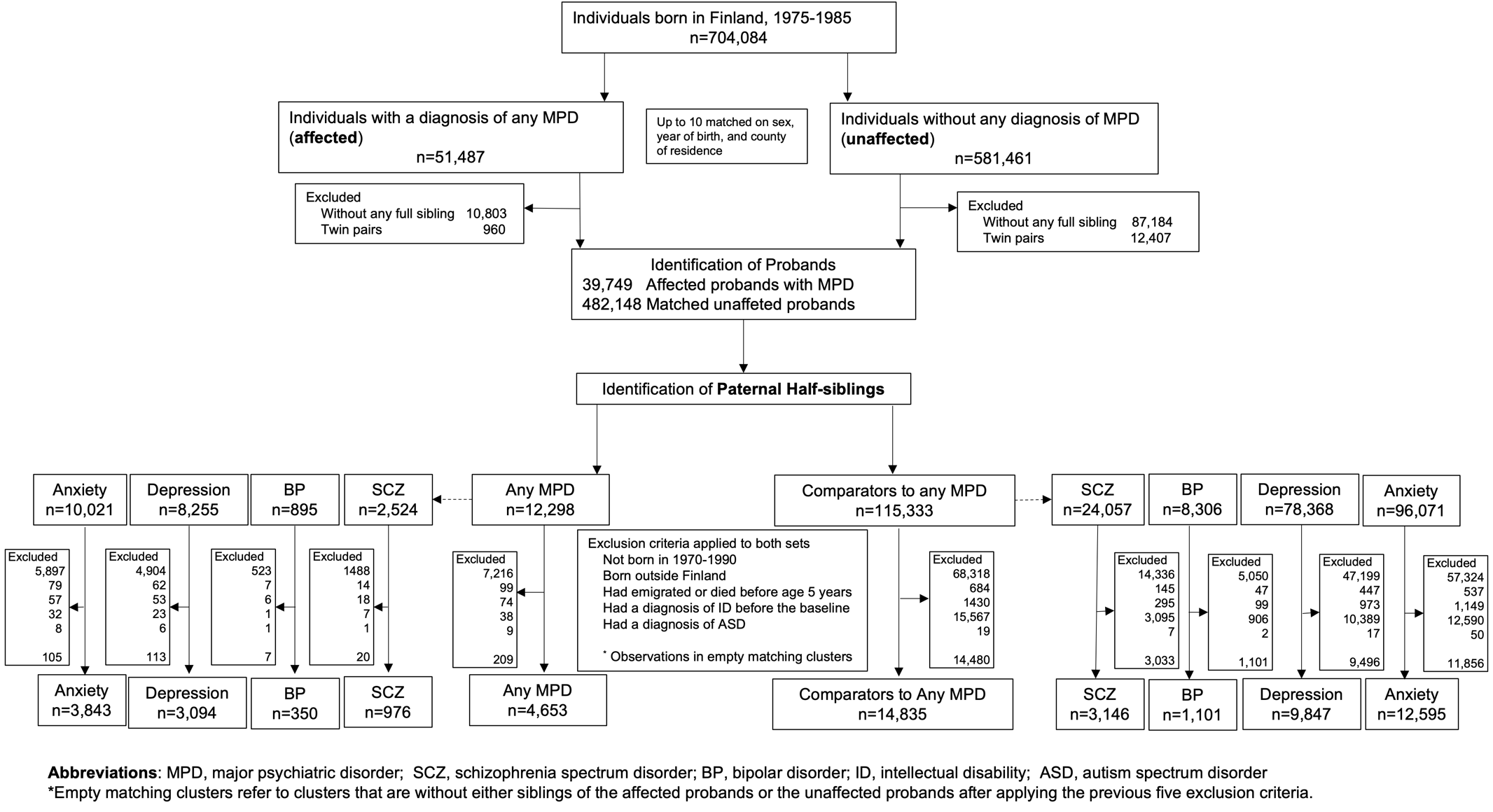
**
